# Supplementary material for: Cross-scanner and cross-protocol multi-shell diffusion MRI data harmonization: Algorithms and results
Source: Neuroimage. Author manuscript; Available in PMC 2023 Jun 6. (PMC10243465; doi:10.1016/j.neuroimage.2020.117128)
Supplement: appendix S1 [file NIHMS1901104-supplement-appendix_S1.pdf]

Cross-scanner and cross-protocol multi-shell diffusion MRI data harmonization: algorithms and results

Lipeng Ning[1,2,*], Elisenda Bonet-Carne[3], Francesco Grussu[3], Farshid Sepehrband[4], Enrico Kaden[3], Jelle Veraart[5], Stefano B. Blumberg[3], Can Son Khoo[3], Marco Palombo[3], Iasonas Kokkinos[3], Daniel C. Alexander[3], Jaume Coll-Font[6,2], Benoit Scherrer[6,2], Simon K. Warfield[6,2], Suheyla Cetin Karayumak[1,2], Yogesh Rathi[1,2], Simon Koppers[7], Leon Weninger[7], Julia Ebert[7], Dorit Merhof[7], Daniel Moyer[4], Maximilian Pietsch[8], Daan Christiaens[8], Rui Azeredo Gomes Teixeira[8], Jacques-Donald Tournier[8], Kurt G. Schilling[9], Yuankai Huo[10], Vishwesh Nath[10], Colin Hansen[10], Justin Blaber[10], Bennett A. Landman[9,10,11], Andrey Zhylka[12], Josien Pluim[12], Greg Parker[13], Umesh Rudrapatna[13], John Evans[13], Cyril Charron[13], Derek K. Jones[13,14], and Chantal M.W. Tax[13]

[1] Brigham and Women's Hospital, Boston, United States
[2] Harvard Medical School, Boston, United States
[3] University College London, London, United Kingdom
[4] Stevens Neuroimaging and Informatics Institute, Keck School of Medicine of USC, University of Southern California, Los Angeles, United States
[5] New York University, New York, NY, United States
[6] Boston Children's Hospital, Boston, United States
[7] RWTH Aachen University, Aachen, Germany
[8] King's College London, London, United Kingdom
[9] Institute of Imaging Science, Vanderbilt University, Nashville, TN, United States
[10] Department of Electrical Engineering & Computer Science, Vanderbilt University, Nashville, TN, United States
[11] Department of Biomedical Engineering, Vanderbilt University, Nashville, TN, United States
[12] Eindhoven University of Technology, Eindhoven, Netherlands
[13] Cardiff University Brain Research Imaging Centre (CUBRIC), Cardiff University, Cardiff, United Kingdom
[14] School of Psychology, Australian Catholic University, Melbourne, Australia

Abstract

Cross-scanner and cross-protocol variability of diffusion magnetic resonance imaging (dMRI) data are known to be major obstacles in multi-site clinical studies since they limit the ability to aggregate dMRI data and derived measures. Computational algorithms that harmonize the data and minimize such variability are critical to reliably combine datasets acquired from different scanners and/or protocols, thus improving the statistical power and sensitivity of multi-site studies. Different computational approaches have been proposed to harmonize diffusion MRI data or remove scanner-specific differences. To date, these methods have mostly been developed for or evaluated on single b-value diffusion MRI data. In this work, we present the evaluation results of 19 algorithms that are developed to harmonize the cross-scanner and cross-protocol variability of multi-shell diffusion MRI using a benchmark database. The proposed algorithms rely on various signal representation approaches and computational tools, such as rotational invariant spherical harmonics, deep neural networks and hybrid biophysical and statistical approaches. The benchmark database consists of data acquired from the same subjects on two scanners with different maximum gradient strength (80 and 300 mT/m) and with two protocols. We evaluated the performance of these algorithms for mapping multi-shell diffusion MRI data across scanners and across protocols using several state-of-the-art imaging measures. The results show that data harmonization algorithms can reduce the cross-scanner and cross-protocol variabilities to a similar level as scan-rescan variability using the same scanner and protocol. In particular, the LinearRISH algorithm based on adaptive linear mapping of rotational invariant spherical harmonics features yields the lowest variability for our data in predicting the fractional anisotropy (FA), mean diffusivity (MD), mean kurtosis (MK) and the rotationally invariant spherical harmonic (RISH) features. But other algorithms, such as DIAMOND, SHResNet, DIQT, CMResNet show further improvement in harmonizing the return-to-origin probability (RTOP). The performance of different approaches provides useful guidelines on data harmonization in future multi-site studies.

Keywords: Multi-shell Diffusion MRI; Harmonization; Spherical harmonics; Deep learning; Regression.

*Corresponding author: Lipeng Ning
Telephone: 617-525-6024
Fax: 617-525-6214
Email: lning@bwh.harvard.edu

1. Introduction

Diffusion magnetic resonance imaging provides information to characterize tissue microstructure by probing the diffusive displacements of water molecules. It is increasingly used to investigate the structural connections in human brains and tissue abnormalities related to disorders (Kubicki et al. 2007; Pasternak et al. 2018; Mueller et al. 2005). Recent advances in acquisition protocols with multiple b-values provide imaging measures that are even more sensitive and/or specific than standard approaches based on single b-value data (Jensen et al. 2005; Özarslan et al. 2013; Ning et al. 2015). On the other hand, dMRI scans have intrinsic variability caused by various factors including but not limited to scanner field and gradient strength and acquisition protocols (Vollmar et al. 2010; Grech-Sollars et al. 2015; Veenith et al. 2013; Landman et al. 2011). In particular, the variability of imaging measures between datasets acquired from different scanners with different protocols could be much more significant than the intra-scanner variability between two data repetitions acquired from the same subject using the same protocol at a different time (Karayumak et al. 2019). Therefore, it is imperative to reduce the cross-scanner and cross-protocol variability in order to reliably aggregate multi-site databases for increasing statistical power and sensitivity of studies.

In view of the significance of cross-scanner and cross-protocol variability, several types of methods have been recently developed to reliably combine dMRI datasets in multi-site studies (Karayumak et al. 2019; Moyer et al. 2019; Blumberg et al. 2018, 2019; Mirzaalian et al. 2018; Fortin et al. 2017; Mirzaalian et al. 2016; Pohl et al. 2016). Some approaches are able to enhance the spatial or angular resolution or other imaging features of dMRI data with low-b values to match the data quality of the state-of-the-art scanners and protocols (Grech-Sollars et al. 2015; Zhu et al. 2011; Vollmar et al. 2010). However, these approaches were designed or implemented to retrospectively harmonize the statistical differences in imaging measures between groups of subjects from different sites with matched age, gender, handedness and other socio-economic factors. A more ideal approach to evaluate cross-scanner and cross-protocol variability is to use datasets from the different scanners of the same subjects. This enables accurate measurement of the intra-subject reproducibility and cross-scanner variability that is not provided by retrospective analysis using group data. In recent years, the intra-scanner reproducibility has been investigated using test and retest scans from the same subjects using the same scanner (Shahim et al. 2017; Duan et al. 2015; Boekel et al. 2017) or using scanners with different field strengths (1.5T vs 3T and 3T vs 7T). We here specifically seek to understand scanner and/or protocol related variability in current multi-site studies based on 3T scanners.

To facilitate research on cross-scanner and cross-protocol variability, a benchmark database was provided in (Tax et al. 2019) with data acquired from 3 different scanners using 5 different acquisition protocols. An open competition was organized during the Computational Diffusion MRI (CDMRI) workshop at 20th International Conference on Medical Image Computing and Computer Assisted Intervention (MICCAI 2017) which invited participants to develop algorithms to solve a dMRI harmonization task which is to find a mapping between datasets acquired using different scanners and/or protocols that were acquired with the same b-value. The result of the competition was reported in (Tax et al. 2019). Most algorithms, e.g. (Koppers et al. 2018), used spherical harmonics to represent single-shell dMRI signals and applied convolutional neural networks (CNNs) to learn mapping between datasets.

While the majority of previous works focused on the harmonization of single-shell diffusion MRI, this work presents the evaluations of harmonization algorithms for multi-shell diffusion data. In

particular, this paper is a continuation and extension of the results in (Tax et al. 2019), and reports on the "MUlti-SHell Harmonization and enhancement Challenge" (MUSHAC) (Ning et al. 2019), part of CDMRI at MICCAI 2018. We report the summary and results of the second open competition on harmonising multi-shell dMRI datasets. The database used in this competition consists of four acquisitions of the same 15 healthy participants scanned on two MRI systems with different maximum gradient strength (80 and 300 mT/m) and different protocols. Different from the earlier work that focused on single-shell data, this paper reports the results of multi-shell comparison from a significantly wider range of algorithms (19 algorithms from 9 research groups). Such systematic evaluations (Klein et al. 2009) have the potential to provide important guidelines for further multi-site studies on diffusion MRI.

Results demonstrate that state-of-the-art computational tools, e.g. CNN and bio-statistical approaches, enable accurate harmonization of multi-shell diffusion MRI across scanners and protocols, reducing between-scanner/between-protocol variability to levels comparable to scan-rescan variability. These results pave the way towards reliable combination of multi-center data in large clinical study, which is key in the area of big data.

2. Methods

The acquisition parameters and preprocessing approaches are described in Section 2.1. Section 2.2 introduces the tasks and rules of the MUSHAC challenge on multi-shell dMRI harmonization. Section 2.3 provides the evaluation strategies and metrics. Section 2.4 summarizes the algorithms participating in the open competition.

2.1. Data

The database was acquired from 15 healthy volunteers, which includes all the subjects reported in Table 1 of (Tax et al. 2019). The data acquisition was approved by Cardiff University School of Psychology ethics committee. All subjects were scanned using three different scanners with different maximum gradient strength. This work focuses on the multi-shell database acquired from the two scanners with relative higher maximum gradient strength: a) 3T Siemens Prisma (80 mT/m), and b) 3T Siemens Connectom (300 mT/m). The average time between acquisitions on scanners a) and b) was 1 month. The scanners had no software upgrades during the course of the study.

Two types of protocols were acquired from each scanner: 1) a more 'standard' (ST) protocol with acquisition parameters matched to a typical clinical protocol; and 2) a more advanced or 'state-of-the-art' (SA) protocol where the superior hardware and software specifications were utilized to increase the number of acquisitions and spatial resolution per unit time. The ST data from both scanners have an isotropic resolution of 2.4 mm, TE = 89 ms and TR = 7.2 s. Both ST datasets have 30 directions at b = 1200, 3000 s/mm^2. On the other hand, the Prisma-SA data has a higher isotropic resolution of 1.5 mm, TE = 80 ms, TR = 7.1s and 60 directions at the same b-values while the Connectom-SA data has the highest resolution of 1.2 mm with TE = 68 ms, TR = 5.4 s and 60 directions. Reversed phase encoding, i.e. AP and PA, b0 images were acquired in both protocols. Additional b=0 s/mm^2 images were also acquired with matched TE and TR across scanners. Moreover, structural T1-weighted (T1w) images were acquired on both scanners using an MPRAGE sequence with 1 mm^3 isotropic voxel. More detailed information about the acquisition parameters can be found in Table 2 of (Tax et al. 2019).

The data preprocessing approach includes the following step: The b0 volumes were corrected for EPI distortions by applying FSL TOPUP on reversed phase-encoding pairs (Andersson et al. 2003). The data was corrected for eddy current induced distortions and gradient-nonlinearity distortions (Glasser et al. 2013) with FSL TOPUP/eddy and in-house software kindly provided by Massachusetts General Hospital (MGH) (Andersson and Sotiropoulos 2016). Spatiotemporally varying b-vectors and b-values due to gradient nonlinearities of the Connectom scanner were made available (Bammer et al. 2003; Sotiropoulos et al. 2013; Rudrapatna et al. 2018). The dMRI of Prisma-SA and Connectom-SA were affinely registered to Prisma-ST using the corresponding fractional anisotropy (FA) maps with FSL FLIRT (Jenkinson and Smith, 2001) and using sinc interpolation and appropriate b-vector rotation. Prisma-ST was used as the reference space since additional geometric distortions can still be present in Connectom data because of gradient nonlinearities (Setsompop et al. 2013; Jones et al. 2018; Tax et al. 2019). The T1w images were registered to the FA of Primsa-ST to avoid extra interpolation of the dMRI data.

2.2. Competition tasks

The processed data from 10 randomly selected subjects were used as training datasets which were released to the participants of the MUSHAC. The Prisma-ST scans and the corresponding T1w images of the remaining 5 subjects were used as a testing set. The tasks were to predict the other three datasets using the provided Prisma-ST scans. In particular, the following two tasks were evaluated:

1) **Harmonization:** the prediction of dMRI signals of the Connectom-ST dMRI scans using the provided Prisma-ST data;
2) **Enhancement:** the prediction of dMRI signals of the state-of-the-art dMRI scans, including Prisma-SA (Task 2a) and Connectom-SA (Task 2b) scans, using the provided Prisma-ST data.

All participants of the dMRI harmonization competition were required to at least complete Task 1, but Task 2 was optional. The preliminary results of the 12 algorithms were announced during the CDMRI workshop at MICCAI 2018 (Ning et al. 2019), as this provided useful feedback regarding the algorithm performance and capabilities to the participants. Then, the participants were allowed to re-submit their updated results or new results after the workshop. New participants were also allowed to enter the competition without providing preliminary evaluations of their algorithms. By the final deadline of the competition, 19 algorithms implemented by 9 different research groups were submitted for evaluation on the test datasets.

2.3. Evaluation

Harmonization algorithms had to predict the dMRI signals of three missing datasets by using the provided b-values, b-vectors and the Prisma-ST scans. From the predicted and the underlying ground-truth datasets, diffusion tensor models were estimated using the dMRI signal at b = 0, 1200 s/mm^2 using a weighted least-squares estimate provided by the dtifit command in FSL (Jenkinson et al. 2012). The fractional anisotropy (FA) and mean diffusivity (MD) measures were subsequently extracted for each voxel. Moreover, two multi-shell dMRI measures, including the mean kurtosis (MK) provided by the diffusion kurtosis imaging (DKI) model (Jensen et al. 2005) and the return-to-origin probability (RTOP) measure from MAP-MRI (Özarslan et al. 2013), were

computed using multi-shell diffusion data. The FA, MD, MK and RTOP measures were all computed using the Dipy toolbox (Garyfallidis et al. 2014). In addition, the zeroth and second order rotationally invariant spherical harmonic (RISH) features, R0 and R2 (Mirzaalian et al. 2016), were computed for the normalized dMRI signals at b = 1200, 3000 s/mm2 to measure the angular frequency of dMRI signals at each b-value shell. The RISH features were computed using an in-house MATLAB (Mathworks, Natick, MA) toolbox.

The estimated imaging measures of the predicted datasets were compared against the corresponding features derived from the true acquired data. The performances of algorithms were evaluated in brain regions specified by brain masks excluding the cerebellum, which was not always included within the foot-head field-of-view coverage. Such an evaluation mask was obtained with the Geodesic Information Flow (GIF) algorithm (Cardoso et al. 2015), which was used to segment different brain areas in the anatomical T1w images. The evaluation masks were provided to the participants. Then, the absolute-value of the percentage error (APE) between the predicted and ground-truth measures, i.e. APE = |(predicted – ground-truth)/ground-truth|, were computed on a voxel level. Subsequently, the mean and standard deviation of APE were computed globally in a brain mask excluding cerebellum, and regionally in brain regions provided by FreeSurfer (Fischl 2012).

2.4. Algorithms

Participants of the open competition developed 19 algorithms to solve the harmonization challenge (Task 1: the prediction of the Conectom-ST data using the Prisma-ST data). A subset of 19 algorithms were also implemented to solve the enhancement challenge (Task 2: the prediction of Prisma-SA data (Task: 2a) and Connectom-SA data (Task: 2b) using the Prisma-ST data). For Task 1, the Prima-ST and the Connectom-ST scans have exactly the same acquisition parameters, including the spatial resolution and the gradient directions. Therefore, the voxel-wise differences in imaging features between the two datasets can be directly computed without using any interpolation algorithms. The corresponding APE value reflects the true cross-scanner differences which can be considered as the ***reference*** when evaluating the performance of harmonization algorithms since no harmonization algorithms were applied. Since the voxel size of Prisma-ST scans are different from those of Prisma-SA and Connectom-SA scans, no natural reference is available for Task 2a and Task 2b. But the performance of interpolation-based algorithms can be considered as the baseline to evaluate other more complex algorithms. A detailed description of the participating algorithms is provided below and a brief summary is presented in Table 1.

2.4.1. Spherical-harmonic based interpolation (SHInterp)

Additional nonlinear registrations based on ANTs (Avants et al. 2011) were applied to align the Prisma-ST datasets to the other three scans with B-spline interpolation methods. Then the MPRAGE images were applied to obtain brain segmentations using the FSL FAST (Jenkinson et al. 2012) and to obtain brain masking using FSL BET command (Jenkinson et al. 2005). The normalized diffusion signals in white matter (WM) and gray matter (GM) voxels within the brain mask were transformed to SH coefficients with order four and Laplace-Beltrami regularization of $\lambda = 0.006$.

The *SHInterp* approach simply transformed the SH coefficients back to diffusion signals along the set of gradient directions of the to-be-predicted scans. Thus, due to SH fitting, it is analogous to a spatial smoothing or regularization in the angular domain of each shell. Since this approach didn't remove any variability in the data, its performance is expected to reflect the true cross-scanner and/or cross-protocol variability of the datasets. For Task 2, standard cubic interpolation was utilized to increase the spatial resolution.

Table 1: *Summary of harmonization algorithms evaluated*

Type	Algorithm name	Additional processing	Signal representation	Algorithm details	Tasks
Interpolation	SHInterp	Nonlinear registration, 4th order SH	SH	Laplace-Beltrami regularization, cubic interpolation	1, 2a, 2b
	DIAMOND-a<br>DIAMOND-b	Noise filtering	DIAMOND	Sinc interpolation,<br>central and non-central DIAMOND models, constrained estimation	1, 2a, 2b
	SHOREInterp	Interpolation	SHORE	Signal prediction using SHORE model	1, 2a, 2b
Regression	LinearRISH	unringing, 6th order SH	SH	Subject-specific training data	1, 2a, 2b
	TWRF-a<br>TWRF-b	Tissue segmentation	DTI<br>Mean signals	Random forest regression with full and reduced feature sets	1
Convolutional Neural Networks	SH-1Layer	Interpolation, 4th order SH	SH	A single convolutional layer	1, 2a, 2b
	SHResNet-a	Nonlinear registration, tissue segmentation	SH	ResNet, Anatomical constraints	1, 2a, 2b
	SHResNet-b0<br>SHResNet-b1<br>SHResNet-b2<br>SHResNet-b3<br>SHResNet-b4	Interpolation, 4th order SH	SH	ResNet,<br>One-hot orthogonal vectors of tissue labels,<br>Spatial coordinates	1, 2a, 2b
	SphericalNet	Nonlinear registration, tissue segmentation	SH	Local Spherical Convolution Network,<br>Anatomical constraints,	1, 2a, 2b
	Tinypatch	Tissue segmentation	SH	Patch-wise feed forward with auto-encoder pre-training	1, 2a, 2b
	DIQT	4th order SH	SH	Patch selection based on center voxels	1, 2a, 2b
	CMResNet-a<br>CMResNet-b	Unringing, inhomogeneity corrections, normalization, nonlinear registration	SHARD	ResNet networks,<br>Two decoder networks	1<br>1, 2a

2.4.2. DIAMOND-based approach (DIAMOND-a -b, -c and -d)

This approach applied two additional preprocessing steps for noise filtering and data up-sampling. In particular, noise filtering was done by using the low-rank matrix approximation approach in (Veraart et al. 2016) followed by correcting for the Gibbs ringing approach (Kellner et al. 2016). The up-sampling approach was applied to interpolate the data with isotropic voxels of size 1 mm using the ITK with "sinc" interpolation (Meijering et al. 1999).

The signal prediction approach was developed based on the hybrid biophysical and statistical DIAMOND method (Scherrer et al. 2016, 2017), which fits a probabilistic model that characterizes diffusion signals with a multi-compartment model. DIAMOND models each compartment in each voxel with a continuous statistical distribution of diffusion tensors (Scherrer et al. 2016), the expectation of which characterizing the average 3-D diffusivity rates of the compartment and the concentration of which capturing the intra-compartment microstructural heterogeneity. The number of fascicle compartments at each voxel was automatically determined using AICU model order selection method (maximum: 3 fascicle compartments). All voxels also included an isotropic diffusion compartment to characterize free-water diffusion.

Two algorithms, denoted by DIAMOND-a and DIAMOND-b, based on the central DIAMOND (Scherrer et al. 2016, 2017) and the non-central DIAMOND models (Scherrer et al. 2016, 2017) were used to characterize diffusion signals, the latter allowing for the separate modeling of axial and radial heterogeneity. In both algorithms, the sum of model coefficients was constrained to be 1. The participant team also submitted two other algorithms based on central DIAMOND and non-central DIAMOND without the constraint on the sum of coefficients. But their experimental results are very similar to the results of DIAMOND-a and DIAMOND-b. Thus, the results without the constraints are not reported in this work.

Based on the estimated model coefficients using the Prisma-ST dataset, the same signal expressions were applied to predict diffusion signals using the b-vectors and b-values of the other three scans. No additional steps were applied to remove potential variability among the datasets from different scanners and protocols.

2.4.3. SHORE-based interpolation (SHOREInterp)

First, the Prisma-ST scans were interpolated to match the spatial resolution of the other three scans to be predicted. Then the Simple Harmonic Oscillator based Reconstruction and Estimation (SHORE) model (Merlet and Deriche 2013; Cheng et al. 2011) was used to represent the normalized multi-shell diffusion signals. More specifically, SHORE was used at the 4th order and regularized least squares were used for fitting as described in (Merlet et al. 2013; Nath et al. 2019). The regularization hyper-parameters were both set to 1e-8 and 'zeta' at 700 (mm^{-2}) as suggested in (Merlet et al. 2013; Nath et al. 2019). The model parameters estimated using the Prisma-ST datasets were used to reconstruct diffusion signals of the other three scans using the respective b-values and b-vectors. No other approaches were used to further reduce potential variability across scans.

2.4.4. Linear regression based on RISH features (LinearRISH)

This approach, denoted by LinearRISH, first preprocessed the raw diffusion MRI following the HCP pipeline steps using an in-house software. Later, the Gibbs unringing approach (Kellner et al. 2016) was applied to reduce the noise in the Prisma-ST data. Then, the steps described in

(Karayumak et al. 2019) were followed: (i) RISH features upto 6th order were estimated separately for diffusion signals at two b-shells with b=1200, 3000 s/mm^2, respectively, using a Tikhonov regularization based algorithm for estimating SH coefficients with the regularization parameter being 0.001; (ii) The mean templates were created for each RISH feature at b = 3000 s/mm^2 using the antsMultiVariateTemplateConstruction2.sh command provided by ANTs (Avants et al. 2011); (iii) The scale maps that characterize the corresponding cross-scanner and/or cross-protocol variability were calculated between the mean RISH features from the Prisma-ST datasets and the other three scans. Consequently, the estimated maps were applied to scale the SH coefficients in the subject space. The same steps were also applied to scale the SH coefficients corresponding to b = 1200 s/mm^2; (iv) The scaled SH coefficients were transformed back to diffusion signals along the provided set of b-vectors for the two b-shells, respectively and the harmonized signal at each shell was estimated in this way. Finally, to preserve the overall integrity of the harmonized signal, the mean harmonized diffusion signal is scaled with the average signal from the other three protocols.

Considering potential inter-subject variability and the limited number of training subjects, the adaptive LinearRISH approach used different subsets of the training dataset to estimate mappings for each test subject. Briefly, from the training dataset, heuristically, the three most similar subjects to each test subject were selected. The similarity criterion was based on the mean squared error of the RISH features of the Prisma-ST dataset and test subjects in the whole-brain as well as region-wise level. The LinearRISH steps described above were followed using subsets of the training dataset to harmonize each test subject.

2.4.5. Tissue-wise regression forest (TWRF-a and TWRF-b)

The MRPAGE images were applied to obtain label maps for WM, GM and CSF regions using FSL FAST (Zhang et al. 2001). For each tissue region, a random forest regression (Breiman 2001) was trained to predict diffusion signals using different training features. Two sets of training features were extracted for each voxel. The first set included the normalized diffusion signal, the eigenvalues of diffusion tensor, the trace, linearity, planarity and sphericity, which were selected to make the solution comparable to (Alexander et al. 2017), and mean signals from the 3x3x3 neighbor voxels, b-values, the b-vectors of both input and the to-be-predicted scans. The second set of features only included the normalized diffusion signals and the mean of the neighborhood. The following parameters were used in the training: the max depth of the decision trees = 10, the number of decision trees (estimators) = 10, criterion was mean squared error, allowed samples per leaf nodes = 1. The *scikit-learn* toolbox (Pedregosa et al. 2011) was used for the training. The prediction results corresponding to the two methods were denoted by TWRF-a (full feature set) and TWRF-b (reduced feature set), respectively. As for training, 6 subjects were used to train the algorithm and the rest subjects were used for validation, implementing the algorithms to the testing datasets.

2.4.6. Single-layer SH network (SH-1Layer)

This approach first interpolated Prisma-ST to match the resolution of the two SA scans. Then, the 4th order SH coefficients of the normalized diffusion signals, i.e. s(b, **u**)/s(0) with s(b, **u**) being the diffusion signal with b-value = b along the gradient direction **u** and s(0) being the baseline, were computed separately at the two non-zero b-shells. The harmonization algorithm was based on a network with a single convolutional layer which took the SH volume (both shells concatenated) as input and returns a spherical harmonic volume (again, both shells concatenated) as output. It was trained without validation because the relative lower risk of overfitting for this simple network. The performance of SHNet can be considered as a “baseline” to which other more complex

network-based methods should be compared. The network was trained using the Adam optimizer and the mean-squared error (MSE) as the loss function with the learning rate being 0.0001 and the number of epochs being 30.

2.4.7. SH based ResNet networks (SHResNet)

Six methods from two participating teams were developed based on similar ResNet structures (Koppers et al. 2018) using concepts from (He et al. 2016) to learn mappings between SH volumes.

SHResNet-a: The method from the first team applied the same preprocessing steps as SHInterp. This algorithm, denoted by SHResNet-a, used the SH coefficient from a 3x3x3 voxel neighborhood (input size: 3x3x3x15) to predict the 15-dimensional SH coefficients in the center voxel using 3D-convolutional networks. The predicted coefficients were converted to diffusion signal using the inverse SH transform. A more detailed information on the structure of this network can be found in Figure 3 (Tax et al. 2019).

SHResNet-b: The methods from the second team includes five variants, i.e. SHResNet-b0 to -b4, that all applied the same preprocessing approaches as SH-1Layer. The first approach, denoted by *SHResNet-b0*, used the same network structure and training method as *SHResNet-a*. The difference between SHResNet-a and SHResNet-b0 is related to the differences in preprocessing approaches, i.e. nonlinear registration v.s. interpolation. Methods *SHResNet-b1, SHResNet-b2,* and *SHResNet-b3* included a one-hot orthogonal vector of SLANT labels (Huo et al. 2018) as input to reduce anatomically dependent variability across scanners. In particular, SHResNet-b1 used 133 labels to denote different anatomical regions. SHResNet-b2 simplified the hierarchy of the labels into 23 unique regions, (i.e. combine cortical lobes, combine cerebral WM, cerebellum, etc.). SHResNet-b3 further smoothed the labels using a Gaussian filter. Instead of using anatomical labels, Method SHResNet-b4 included a one-hot orthogonal vector of voxel location in x, y, and z coordinates (normalized from -1 to 1) as input for reducing FOV/location dependent artifact. All the models, SHResNet-b0 to SHResNet-b4, were trained using the same parameters and cost functions as SH-1Layer in Section 2.4.6.

2.4.8. Spherical network (SphericalNet)

This method combines the novel local spherical convolution layer (LSC) (Koppers and Merhof 2018) with the SHResNet (Koppers et al. 2018). The LSC layers are utilized as pre- and postprocessing layer of the network. First, it separates the two input-shells into ten linear combinations, based on the neighboring q-space signals, while the last layer combines the output of the ResNet to predict the two shells of the three to-be-predicted scans. Further, each residual block was extended to be applicable to multi-shell data.

2.4.9. Tiny-patch network (Tinypatch)

In pre-processing, this method, named Tinypatch, applied FSL FAST to obtain label maps for white-matter, grey-matter, and CSF regions using the MPRAGE images. The diffusion data were projected to an 8th order Spherical Harmonics (SH) basis, using an L2-minimal projection. These projections were concatenated voxelwise with the tissue labels and then fed into a patch-wise feed-forward fully connected neural network, which output estimates of a corresponding patch from the to-be-predicted site.

Patches were constructed using a center voxel and the 6 immediately adjacent voxels. The input was then vectorized, and then fed into the fully connected network. The neural network consisted of 2 encoder layers, one center layer, and 2 decoder layers, with (128,64), (32), and (64,128) hidden units respectively, using tanh non-linear activations at each hidden unit. The encoder and center layers were pre-trained using an auto-encoder task, attempting to reconstruct the base site using a temporary set of decoder layers. The full network was then trained using corresponding patches between scans; for the higher resolution scans (Task 2a and Task 2b) the spatial offsets were concatenated to the activations at the center layer. Loss was computed by projecting SH estimates back to the subject specific b-vectors. This loss was then propagated back to the rest of the network.

A separate network was trained for each task site (Task 1, Task 2a, and Task 2b). Each network was trained to convergence on 9 of the training subjects, leaving one subject for validation. The best performing weight set on the validation subject was then run on the test dataset, and the outputs submitted for evaluation.

2.4.10. Deeper image quality transfer (DIQT)

Raw data were pre-processed to extract signal features by spherical harmonics (SH) deconvolution (Tournier et al. 2004). The pre-processing was performed using an home-made python script, also including some functions from DIPY (Garyfallidis et al. 2014). For each of the 2 diffusion gradient shells, the 15 coefficients of the 4th order SH deconvolution were estimated from the normalized raw signal at different diffusion gradients directions, using real and symmetric SH basis. These 30 SH coefficients (15 coefficients of the 4th order SH deconvolution per each of the 2 shells) computed from the available subjects in the training set, were then used as input to a CNN, to predict the corresponding 30 SH coefficients for the unseen subjects in the test set. From the predicted 30 SH coefficients, the normalized raw signal for the subjects in the test set was computed by evaluating the SH in the provided set of directions.

The DIQT approach used supervised learning on 3D input-target patches that were extracted from the Prisma-ST scans. The center voxel of each input patch was contained within the brain mask, and each patch was of physical size $11x2.4^3$ mm^3. Before extracting the patches, the mean and standard deviation were computed for each input channel, i.e. SH coefficient across training subjects. Then the training (and validation) data was normalized to have zero mean and the standard deviation one. The set of input patches were then used to predict image patches in the target space where the size of the target patch was chosen so that, when considered in the same physical space, the target patches were contained within the input patches.

The performance validation of this algorithm was initially explored on two fixed subjects. Then cross-validation was utilized, where the hold-outs were different to the data exploration subjects, to estimate the generalized error-rate. 10% of the training data was allocated as a pre-validation set, for early stopping of model training. The final validation/submission was performed by multiple reconstruction of a subject's brain. For a reconstruction, a subject's input brain was divided into non-overlapping input patches, where the patch predictions were combined to create a whole brain prediction. This procedure was performed 64 times and the reconstructions averaged. Variants of the DIQT Network (Blumberg et al. 2018; Blumberg et al. 2019) were used for the three tasks, where the shuffle in (Blumberg et al. 2018) was removed in Task1. In Tasks 2 and 3, the shuffle in was replaced with a transposed convolutional layer. The model hyperparameters of these networks, including the number of reversible network layers per block (a proxy for the

network's depth), size of input/target patch, learning rate and decay, sampling scheme for training data, were explored in (Blumberg et al. 2019). The code for the extension of this challenge and (Blumberg et al, 2019) is available at *https://github.com/sbb-gh/*.

2.4.11. Spherical Harmonics and a Radial Decomposition (SHARD) based ResNet (CMResNet-a and CMResNet-b)

As preprocessing, this approach applied the Gibbs unringing toolbox (Kellner et al. 2016) to suppress noise and the ANTs N4 tool (Tustison et al. 2010) to correct for intensity inhomogeneity. Then, the Connectom scans were diffeomorphically registered the Prisma data using multi-modal registration as implemented in MRtrix3 (*mrregister*) (Raffelt et al. 2011), with sum-of-squared differences cost function between FA, MD and histogram-matched b0.

Next, this method used the Spherical Harmonics and a Radial Decomposition (SHARD) proposed in (Christiaens et al. 2018) as rotation-invariant representation of multi-shell dMRI data. SHARD learns the optimal low-rank representation of a given dataset (single subject or group), using a singular value decomposition across shells and spherical harmonic bands. In this work, a groupwise basis was learned for each protocol (*Prisma-ST* and *Connectom-ST*) from the 6 training subjects (subjects A-F). The data of all (training, validation, and test) subjects is then projected onto the basis, thus representing each scan by a 4-D image consisting of 57 volumes of SHARD coefficients in the basis of its protocol. The SHARD images were estimated in the original space of Connectom scans then warped to the space of Prisma data.

Two ResNet networks, denoted by CMResNet-a and CMResNet-b, were used to learn mappings between the SHARD images, where each included an encoder and two decoders. The encoder transforms the source SHARD image to a common embedding. The two decoder networks D_S and D_T transform from embedding to source and target domain, respectively. The networks consist of multiple depth-wise separable convolution layers, residual blocks, and a Squeeze-and-Excitation connection (Hu et al. 2017) that uses channel-wise average pooling inside the brain mask as features for adaptive channel scaling.

The structure of the two networks, CMResNet-a and CMResNet-b are shown in Fig. 1 to Fig. 3. The networks use a multiply connection between input and encoder output and expand the N=57 SHARD volume channels to C=72 channels. The networks were trained using a mean squared loss on the SHARD coefficients with either source or target image as input. The transformed image from the single pass through encoder and decoder was evaluated against the same subject scanned with the other protocol (see Figure 1 a) and b)). Additionally, using two passes through the network, the input image was compared to the prediction given itself as input as shown in Figure 1 c) and d). In the first two training methods the loss was evaluated between domains, in the latter, it was evaluated using a single image from either domain. Chaining E, D_S, E and D_T allows training the network using a cycle-consistency loss (Zhou et al. 2016; Liu et al. 2017) of source images without the need for well aligned matching target data. To avoid learning an identity mapping in this setting, either D_T or D_S are held constant during cycle-consistency training steps and the network is trained simultaneously with paired and unpaired loss.

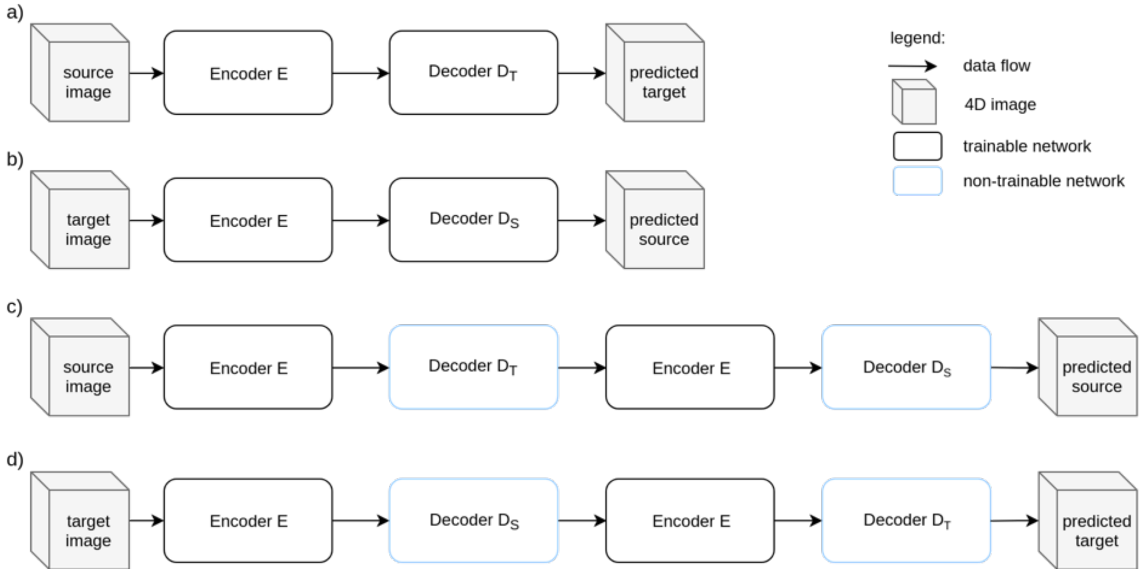

Figure 1: *The network uses the encoder E to transform the source and/or target image to a common embedding that the decoders* D_T *and* D_S *transform to the target and source domain, respectively. The weights are shared across all networks with identical names. The rows show the methods for training the encoder and decoder networks of the networks to predict the target SHARD image given the source SHARD image a), the inverse mapping b), and the full cycle from source to predicted source c) and from target to predicted target d).*

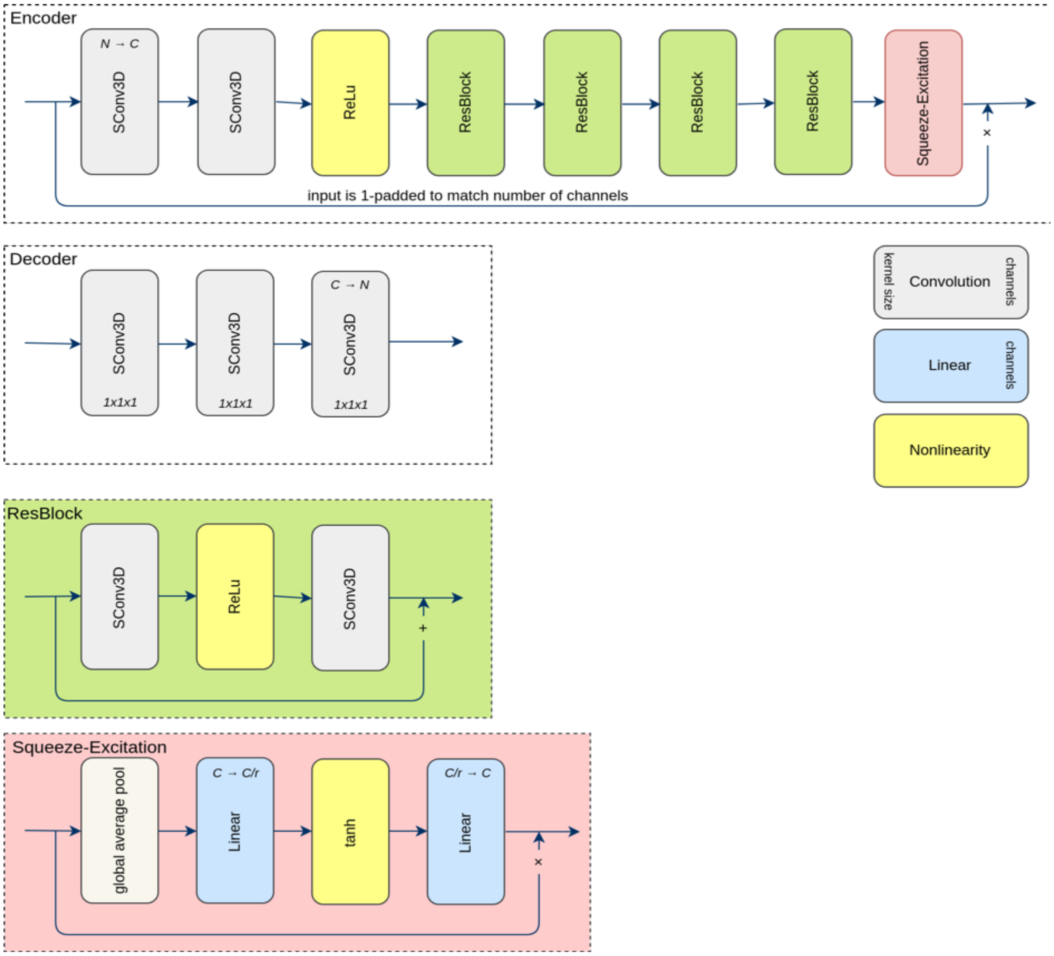

Figure 2: *The encoder and decoder networks architecture of CMResNet-a and their building blocks. SConv3D denotes a depth-wise separable convolution consisting of a convolution (with kernel size 3x3x3, unless otherwise stated, stride=1, zero padded), grouped by input channels, followed by a 1x1x1 convolution (stride=1, no padding) mapping to the number of output channels. The number of channels is omitted if it is unchanged. The Squeeze-and-Excitation reduction factor (r) is 4.*

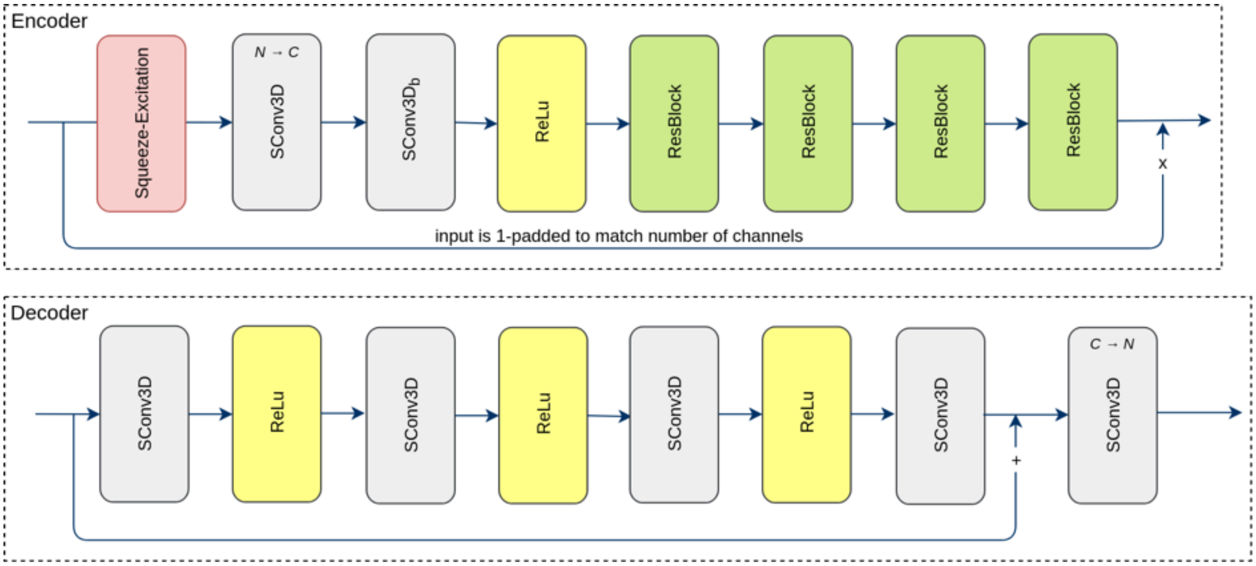

Figure 3: *Encoder and decoder network architecture of the CMResNet-a. See Figure 2 for the layer legend.*

3. Results

3.1. Task 1: matched resolution cross-scanner harmonization

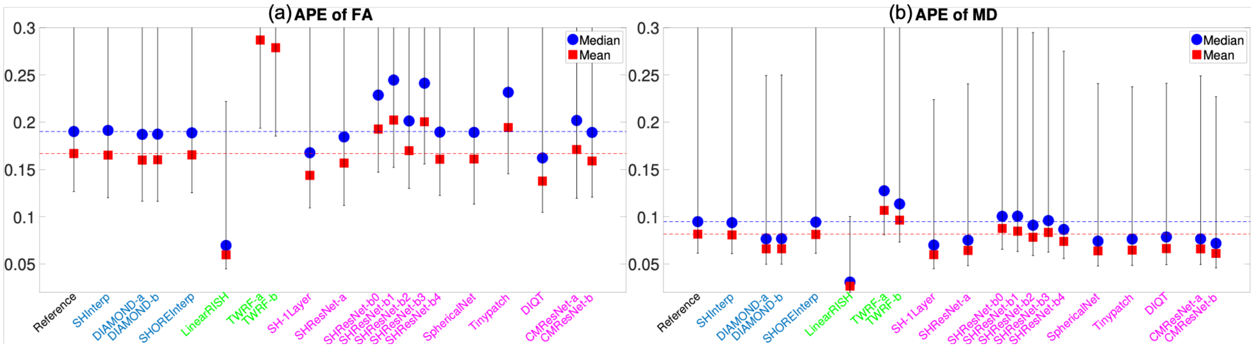

Figure 4: *An illustration of the APE of FA and MD measures computed using diffusion signals at the b-shell with b = 1200 s/mm^2 for algorithms evaluated for Task 1. The reference (dashed lines) illustrates the difference between Prisma-ST and Connectom-ST scans with no harmonization. The y-axis does not include the origin in order to emphasize the differences between methods.*

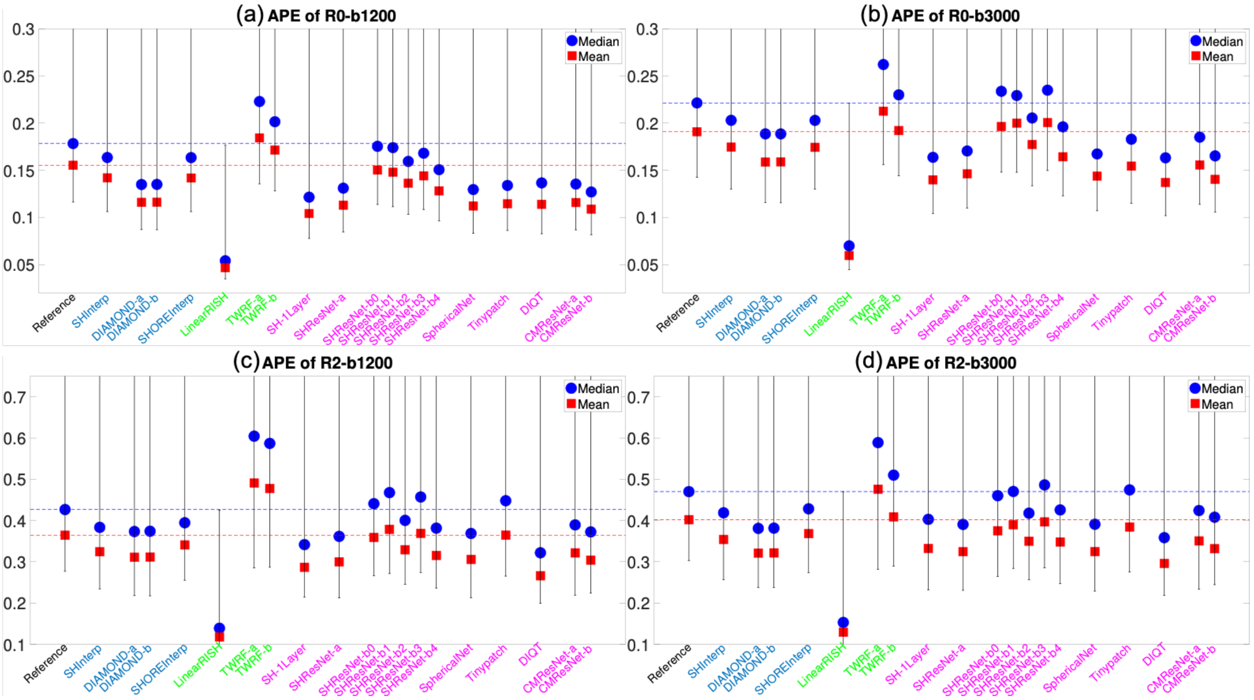

Figure 5: *An illustration of the APE of the zeroth and second order RISH features, R0 and R2, of diffusion signals at two b-shells for the algorithms evaluated for Task 1. The reference (dashed lines) illustrates the difference between Prisma-ST and Connectom-ST scans with no harmonization.*

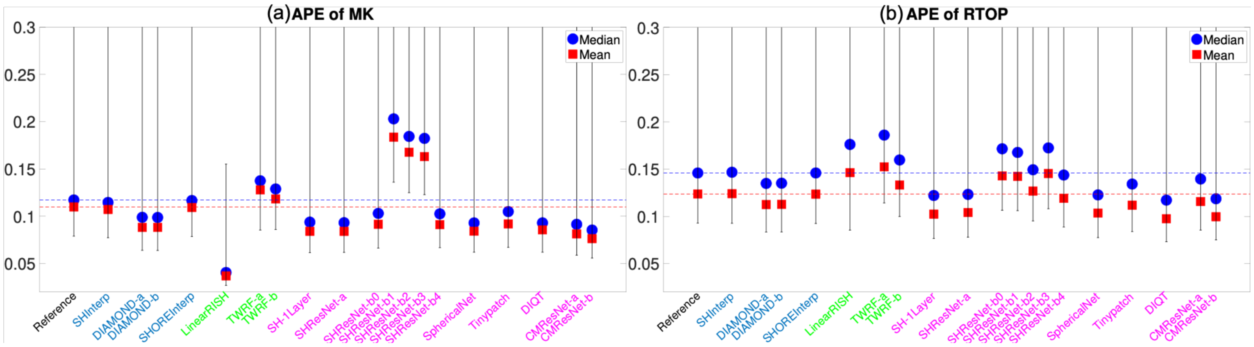

Figure 6: *An illustration of the APE of MK and RTOP computed using multi-shell diffusion signals for algorithms evaluated for Task 1. The reference (dashed lines) illustrates the difference between Prisma-ST and Connectom-ST scans with no harmonization.*

3.1.1. Global evaluation

Figs. 4(a) and 4(b) illustrate the average APE of the FA and MD measures over all voxels within the evaluation masks for 19 algorithms that completed Task 1. These DTI features were computed using only diffusion signals at the b-shell with b = 1200 s/mm^2, since DTI only captures the second order cumulant, and higher-order cumulants become more prominent at higher b-values. Similarly, Figs. 5(a) to 5(d) show the APE of the zeroth and second order RISH features, R0 and R2, for diffusion signals at the two b-shells, respectively. Figs. 6(a) and 6(b) illustrate the APE of MK and RTOP computed using multi-shell diffusion signals. Algorithms of the same type, i.e. interpolation-, regression- or CNN-based, are coded by the same color.

Table 2: *Summary of the percentage APE values, i.e. APEx100%, of reported measures in global evaluation for Task 1.*

	APE (%): Mean (Standard deviation)							
	FA	MD	R0 (b=1200)	R2 (b=1200)	R0 (b=3000)	R2 (b=3000)	MK	RTOP
Reference	16.7 (2.0)	8.2 (1.2)	15.5 (2.0)	36.4 (3.6)	19.1 (2.1)	40.2 (4.3)	11.0 (1.0)	12.4 (1.4)
SHInterp	16.5 (11.2)	8.1 (5.7)	14.2 (10.0)	32.4 (20.8)	17.5 (12.0)	35.4 (22.8)	10.7 (8.7)	12.4 (8.2)
DIAMOND-a	15.9 (10.5)	6.6 (4.6)	11.5 (7.9)	30.9 (18.5)	16.5 (10.1)	32.3 (20.8)	9.1 (6.5)	11.7 (7.2)
DIAMOND-b	15.9 (10.5)	6.6 (4.6)	11.6 (7.9)	30.8 (18.6)	16.4 (10.1)	32.3 (21.1)	9.0 (6.6)	11.5 (7.2)
SHOREInterp	16.5 (12.1)	8.1 (5.7)	14.2 (10.0)	34.1 (23.8)	17.4 (12.0)	36.8 (25.2)	10.9 (8.9)	12.4 (8.2)
LinearRISH	6.0 (4.1)	2.7 (1.8)	4.7 (3.2)	11.8 (7.8)	6.0 (4.0)	12.9 (8.5)	3.7 (2.9)	14.6 (7.2)
TWRF-a	28.7 (15.9)	10.7 (7.0)	18.4 (11.4)	49.1 (22.8)	21.3 (12.5)	47.6 (22.3)	12.8 (9.7)	15.2 (9.4)
TWRF-b	27.9 (15.7)	9.6 (6.6)	17.1 (11.5)	47.8 (22.9)	19.2 (12.4)	40.9 (22.4)	11.8 (9.3)	13.3 (8.5)
SH-1Layer	14.4 (10.0)	6.0 (4.1)	10.4 (7.1)	28.7 (18.6)	14.0 (9.5)	33.2 (19.3)	8.4 (6.3)	10.2 (6.7)
SHResNet-a	15.7 (10.1)	6.4 (4.4)	11.3 (7.9)	30.0 (17.9)	14.6 (10.1)	32.4 (19.6)	8.4 (6.4)	10.4 (6.9)
SHResNet-b0	19.3 (12.9)	8.8 (6.3)	15.0 (10.4)	35.9 (21.5)	19.6 (12.9)	37.5 (21.3)	9.2 (6.7)	14.3 (9.1)
SHResNet-b1	20.2 (12.8)	8.5 (5.6)	14.8 (10.0)	37.9 (21.5)	20.0 (14.1)	39.0 (24.0)	18.4 (14.2)	14.2 (9.5)
SHResNet-b2	17.0 (11.5)	7.8 (5.5)	13.6 (9.4)	32.9 (20.3)	17.7 (12.5)	35.0 (22.2)	16.8 (13.1)	12.7 (8.5)
SHResNet-b3	20.0 (13.3)	8.3 (5.9)	14.4 (10.0)	36.9 (21.7)	20.1 (13.6)	39.7 (23.5)	16.3 (12.5)	14.5 (9.5)
SHResNet-b4	16.1 (11.0)	7.4 (5.1)	12.8 (8.7)	31.5 (19.8)	16.4 (10.6)	34.8 (20.1)	9.1 (6.7)	11.9 (7.5)
SphericalNet	16.1 (10.2)	6.4 (4.4)	11.2 (7.8)	30.6 (18.0)	14.4 (9.9)	32.5 (19.4)	8.4 (6.5)	10.4 (6.8)
Tinypatch	19.4 (12.7)	6.5 (4.3)	11.4 (7.8)	36.5 (21.3)	15.4 (10.2)	38.4 (21.9)	9.2 (6.5)	11.2 (7.2)
DIQT	13.8 (9.4)	6.6 (4.4)	11.4 (7.1)	26.6 (16.7)	13.7 (8.9)	29.6 (18.2)	8.6 (6.8)	9.8 (6.2)
CMResNet-a	17.1 (10.7)	6.6 (4.6)	11.6 (7.9)	32.2 (18.3)	15.6 (10.0)	35.1 (19.5)	8.1 (5.9)	11.6 (7.2)
CMResNet-b	15.9 (10.6)	6.1 (4.1)	10.9 (7.5)	30.4 (18.2)	14.0 (9.5)	33.2 (19.6)	7.6 (5.7)	10.0 (6.5)

The bar plots in Figs. 4 to 6 show the 10th to 90th percentile of the distribution of APE values over all voxels. The blue circle illustrates the median value of APE distributions. The 90th percentile bars are significantly further away from the median values than the 10th percentile bars, indicating that the underlying distributions have long tails which may be driven by outliers. Thus, the mean values, indicated by the red squares, were computed using all the measures lower than the 90th percentile to reduce the effect of outliers, i.e. it is the truncated mean.

The leftmost barplot in Figs. 4 to 6, denoted by "Reference", shows the APE values between imaging measures from the Prisma-ST and Connectom-ST datasets. Since the two datasets have similar scanning parameters and matched spatial resolution, the APE values were computed by voxel-wise differences between the imaging measures. The reference distribution shows the true cross-scanner variability if no harmonization algorithm is applied. The results of a good harmonization algorithm are expected to have lower mean and median APE values than the Reference, which are indicated by the two dashed lines.

The 2nd to 5th barplots in Figs. 4 to 6 show the results of interpolation-based algorithms, i.e. SHInterp to SHOREInterp. It is interesting to point out that all techniques outperformed the reference though the cross-scanner and cross-protocol variability is not considered in the underlying algorithms. In particular, SHInterp and SHOREInterp have similar mean and median values in APE of FA, MD MK and RTOP as the reference. The corresponding APE values of RISH features are slightly lower than the reference, which is possibly due to the denoising effect of the

algorithms. DIAMOND-a and DIAMOND-b have relative lower APE values for MD, RISH features, MK and RTOP than the reference.

The 6th to 8th barplots in Figs. 4 to 6 show the results of regression-based algorithms, i.e. LinearRISH to TWRF-b. The LinearRISH approach has the lowest values in APE of FA, MD, RISH features and MK. But it has a similar APE of RTOP as the reference. On the other hand, TWRF-a and TWRF-b have relative higher APE values than the reference. TWRF-a has worse performance than TWRF-b which may be caused by the redundant features, such as the b-values and the b-vectors.

The last 12 barplots in Figs. 4 to 6, from SH-1Layer to CMResNet-b, illustrate the results based on convolutional neural networks. The results of SH-1Layer serve as a baseline for other deep-network-based results. It can be seen that several deep-network-based algorithms, such as SHResNet-b0 to SHResNet-b3, have much higher prediction error than SH-1Layer, though the underlying network structures are more complex. Other methods, including SHResNet-a, Tinypatch, DIQT, CMResNet-a and CMResNet-b, all have similar performances as SH-1Layer.

A summary of the mean APE values of the 19 algorithms is provided in Table 2. It can be seen that SHInterp has similar or slightly better performance than the reference. The two DIAMOND model-based algorithms have very similar performances, indicating the different choices of DIAMOND models have only little effect on the results. SHOREInterp has similar performances as SHInterp and Reference. TWRF-b is slightly better than TWRF-a, but both actually increased variability compared to the reference. SHResNet-a is the best among the six SHResNet-based algorithms. DIQT is in general the best CNN based approach, but slightly worse than SH-1Layer or CMResNet-b in some measures. Overall, LinearRISH has the best performance in most evaluation metrics but the RTOP measure, in which LinearRISH is the 2nd highest and is worse than the reference and SHInterp.

3.1.2. Regional evaluation

Fig. 7 illustrates the spatial distribution of the mean APE over the ROIs from the Kesikan-Killiany atlas from FreeSurfer (Fischl 2012) in sagittal, axial and coronal views of a brain for 8 representative algorithms. The value in each ROI is the average APE over all measures and all the underlying voxels, with the size of ROIs not considered in the analysis. In particular, Figs. 7(a) to 7(h) show the APE corresponding to Reference, DIAMOND-a, LinearRISH, SH-1Layer, ShpericalNet, SHResNet-b0, DIQT and CMResNet-b. Fig. 7(a) shows that the frontal and temporal lobes have the highest errors, which may be related to residual, uncorrected distortions and motion-induced effects in these regions. The errors are significantly reduced by several methods in Figs. 7(b) to 7(f), though the frontal and temporal lobes still have relatively higher errors than other regions. It can be seen from Fig. 7(c) that LinearRISH has relatively lower errors than other methods in almost all regions.

Table 3 summarizes the mean APE in the gray matter and white matter regions of all 19 algorithms. It also provides the two ROIs with the highest or lowest APE for each method. For all methods, the APE values in gray matter are all higher than the result of white matter. Moreover, several ROIs, such as the entorhinal cortex, the superiorparietal cortex, and the rostralmiddlefrontal cortex, consistently have the highest errors for most algorithms. On the other hand, white matter regions in the paracentral, the banks of the superior temporal sulcus (bankssts) and the insula have the lowest errors.

Table 3: *Summary of the average percentage APE values in GM, WM and two ROIs from the Desikan-Killiany atlas from FreeSurfer with the highest and lowest APE for Task 1.*

	APE (%)		ROIs with the highest APE	ROIs with the lowest APE
	GM	WM		
SHInterp	27.5	17.1	ctx-rh-rostralmiddlefrontal, ctx-rh-superiortemporal	wm-lh-bankssts, wm-rh-insula
DIAMOND-a	23.2	15.1	ctx-lh-entorhinal , ctx-rh-superiorparietal	wm-rh-paracentral, wm-lh-paracentral
DIAMOND-b	23.2	15.0	ctx-lh-entorhinal , ctx-rh-superiorparietal	wm-lh-paracentral, wm-rh-paracentral
SHOREInterp	27.9	17.1	ctx-rh-superiortemporal, ctx-rh-cuneus	wm-lh-bankssts, wm-lh-insula
LinearRISH	10.7	6.7	ctx-rh-superiortemporal, ctx-lh-parsorbitalis	wm-lh-paracentral, wm-lh-bankssts
TWRF-a	26.0	25.5	ctx-lh-entorhinal, wm-rh-parsorbitalis	ctx-lh-isthmuscingulate, ctx-lh-bankssts
TWRF-b	26.2	22.1	ctx-lh-entorhinal, ctx-lh-superiorfrontal	wm-lh-inferiorparietal, wm-lh-lateraloccipital
SH-1Layer	22.0	13.6	ctx-lh-entorhinal, ctx-rh-parsorbitalis	wm-lh-bankssts, wm-rh-paracentral
SHResNet-a	22.8	14.3	ctx-lh-entorhinal, ctx-rh-rostralmiddlefrontal	wm-lh-bankssts, wm-lh-paracentral
SHResNet-b0	30.8	18.6	ctx-rh-parsorbitalis, ctx-lh-parsorbitalis	wm-rh-paracentral, wm-lh-insula
SHResNet-b1	32.6	20.9	ctx-rh-inferiorparietal, ctx-rh-cuneus	wm-lh-bankssts, wm-rh-lateralorbitofrontal
SHResNet-b2	29.2	18.3	ctx-lh-entorhinal, ctx-rh-parsorbitalis	wm-lh-bankssts, wm-lh-insula
SHResNet-b3	31.0	20.4	ctx-lh-entorhinal, ctx-lh-parahippocampal	wm-rh-insula, wm-rh-lateralorbitofrontal
SHResNet-b4	24.0	16.1	ctx-lh-entorhinal, ctx-rh-pericalcarine	wm-lh-paracentral, wm-rh-paracentral
SphericalNet	23.0	14.1	ctx-lh-entorhinal, ctx-lh-parsorbitalis	wm-lh-paracentral, wm-lh-bankssts
Tinypatch	22.8	15.6	ctx-lh-entorhinal, ctx-lh-postcentral	wm-lh-paracentral, wm-rh-lateralorbitofrontal
DIQT	20.9	13.7	ctx-lh-entorhinal, ctx-lh-parahippocampal	wm-lh-bankssts, wm-rh-precuneus
CMResNet-a	23.0	15.4	ctx-lh-entorhinal, ctx-lh-parsorbitalis	wm-rh-paracentral, wm-lh-paracentral
CMResNet-b	21	14	ctx-lh-entorhinal, ctx-rh-rostralmiddlefrontal	wm-lh-paracentral, wm-rh-paracentral

3.2. Task 2: spatial- and angular-resolution enhancement

3.2.1. Global evaluation

The barplots in Figs. 8 to 10 illustrate the APE of 8 measures for 19 algorithms evaluated for Task 2a. Overall, the average APE is higher than the corresponding results in Task 1 shown in Figs. 4 to 6. The reference value of APE is not available in this task since the Prisma-ST and Prisma-SA scans have different spatial resolutions. But the SHInterp approach can be considered as the "baseline" since it provides harmonization errors as assessed in Task 1 that are similar to the intrinsic between-scanner differences. A summary of the mean APE values for 8 evaluation measures is shown in Table 4.

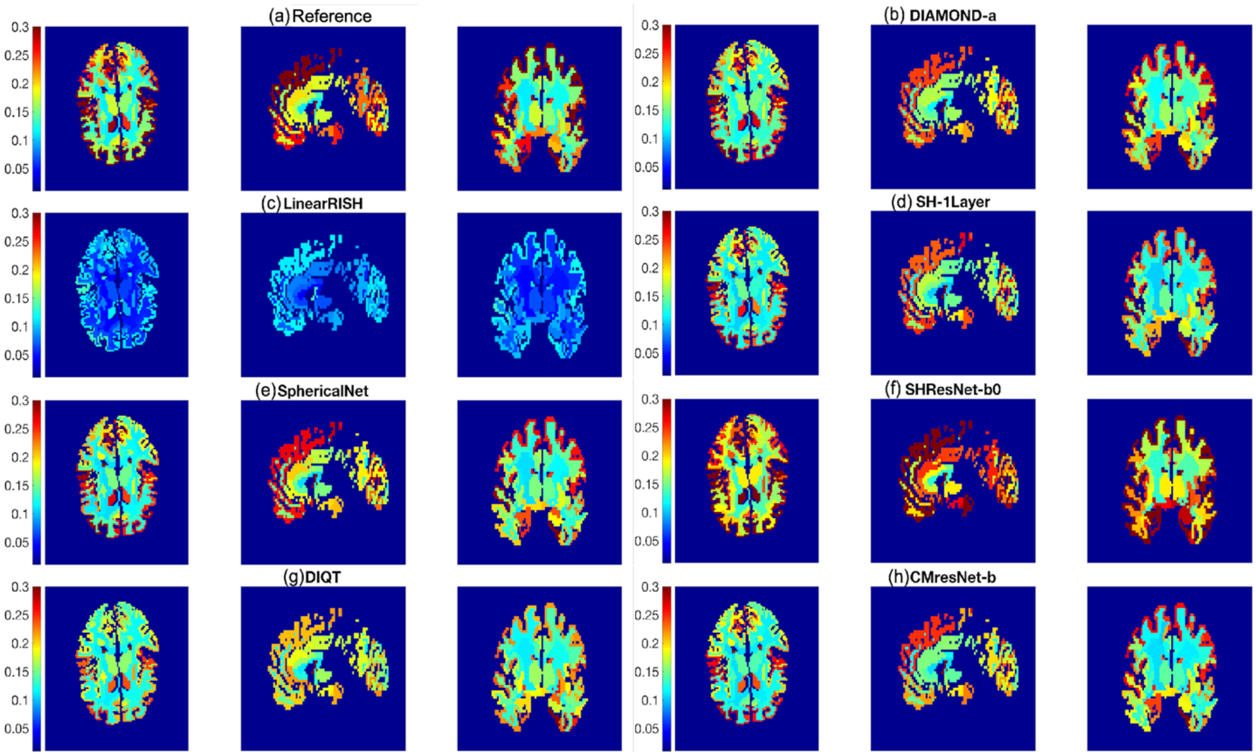

Figure 7: *Illustration of the average regional APE values of all dMRI measures in different brain regions defined by the Desikan-Killiany atlas from FreeSurfer for several representative algorithms evaluated for Task 1 and the reference.*

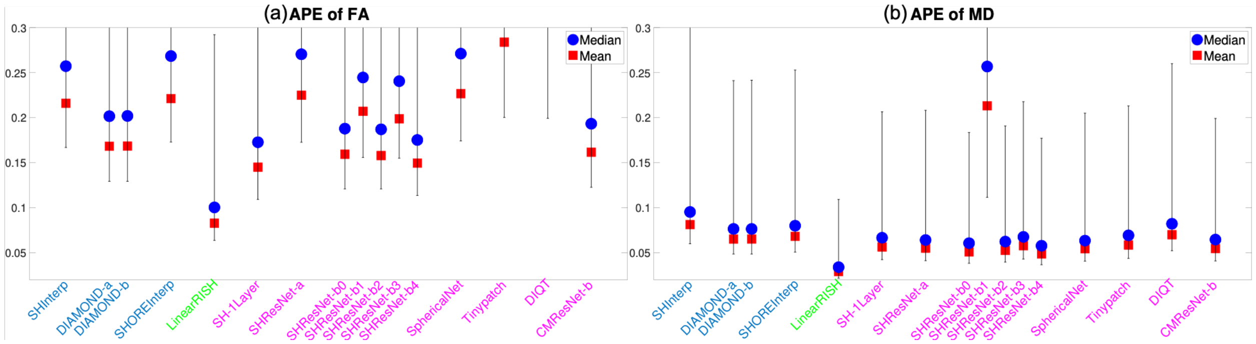

Figure 8: *Global APE values for the FA and MD measures computed using diffusion signals at the b-shell with b = 1200 s/mm^2 for algorithms evaluated for Task 2a.*

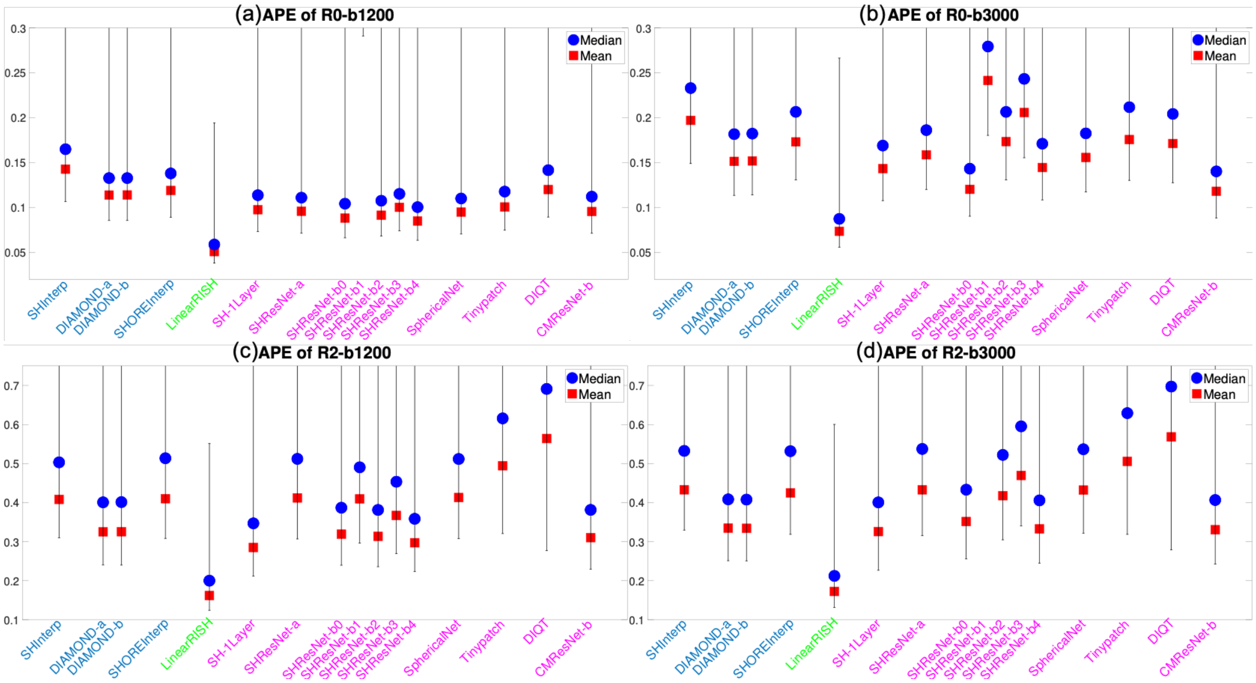

Figure 9: *Global APE values for the zeroth and second order RISH features, R0 and R2, of diffusion signals at two b-shells for the algorithms evaluated for Task 2a.*

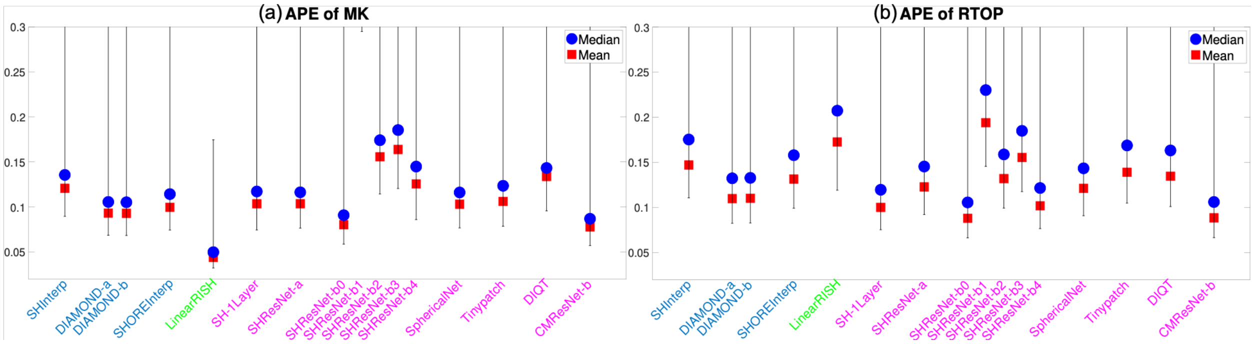

Figure 10: *Global APE values for the MK and RTOP measures computed using multi-shell diffusion signals for algorithms evaluated for Task 2a.*

Table 4: Summary of the percentage APE values, i.e. APEx100%, of reported measures in global evaluation for Task 2a.

	APE (%): Mean (Standard deviation)							
	FA	MD	R0 (b=1200)	R2 (b=1200)	R0 (b=3000)	R2 (b=3000)	MK	RTOP
SHInterp	21.6 (14.5)	8.1 (5.4)	14.3 (10.0)	40.8 (24.8)	19.7 (13.2)	43.3 (26.5)	12.1 (9.0)	14.7 (9.6)
DIAMOND-a	16.8 (11.1)	6.5 (4.4)	11.4 (7.9)	32.6 (19.2)	15.2 (9.7)	33.4 (20.6)	9.3 (6.8)	11.0 (7.0)
DIAMOND-b	16.9 (11.1)	6.5 (4.4)	11.3 (7.8)	32.5 (19.2)	15.2 (9.8)	33.6 (20.8)	9.3 (6.8)	11.0 (7.0)
SHOREInterp	22.1 (14.4)	6.8 (4.6)	11.9 (8.3)	41.0 (23.7)	17.3 (11.3)	42.5 (24.5)	10.0 (7.1)	13.2 (8.5)
LinearRISH	8.3 (5.3)	2.9 (2.0)	5.1 (3.6)	16.2 (9.9)	7.3 (4.9)	17.3 (10.6)	4.4 (3.2)	17.3 (10.1)
SH-1Layer	14.5 (9.5)	5.6 (3.8)	9.8 (6.7)	28.5 (17.5)	14.3 (9.6)	32.6 (18.2)	10.4 (7.4)	10.0 (6.5)
SHResNet-a	22.5 (14.7)	5.5 (3.8)	9.6 (6.7)	41.2 (24.0)	15.9 (10.9)	43.3 (24.7)	10.4 (7.7)	12.3 (8.2)
SHResNet-b0	15.9 (10.8)	5.1 (3.3)	8.8 (5.9)	31.9 (20.0)	12.0 (7.9)	35.1 (20.4)	8.0 (5.8)	8.8 (5.7)
SHResNet-b1	20.7 (13.9)	21.3 (9.5)	47.1 (24.9)	41.0 (25.7)	24.1 (16.9)	288.2 (284.8	46.0 (29.3)	19.4 (12.8)
SHResNet-b2	15.8 (10.7)	5.3 (3.5)	9.1 (6.1)	31.4 (19.5)	17.3 (11.4)	41.8 (23.5)	15.6 (11.6)	13.2 (8.5)
SHResNet-b3	19.9 (13.0)	5.8 (3.9)	10.0 (7.1)	36.7 (21.7)	20.6 (13.8)	47.0 (25.3)	16.4 (11.9)	15.6 (10.3)
SHResNet-b4	15.0 (10.3)	4.9 (3.2)	8.5 (5.6)	29.7 (18.9)	14.5 (9.6)	33.3 (20.1)	12.6 (8.2)	10.2 (6.6)
SphericalNet	22.7 (15.0)	5.4 (3.7)	9.5 (6.6)	41.3 (24.3)	15.6 (10.6)	43.2 (25.2)	10.3 (7.7)	12.1 (8.1)
Tinypatch	28.4 (16.7)	5.9 (3.9)	10.1 (6.8)	49.4 (24.9)	17.6 (11.0)	50.6 (24.8)	10.6 (7.3)	13.9 (8.7)
DIQT	34.3 (16.2)	7.0 (4.7)	12.0 (8.0)	56.4 (22.9)	17.1 (11.1)	56.8 (23.0)	13.4 (10.7)	13.5 (8.4)
CMResNet-b	16.2 (10.6)	5.5 (3.6)	9.5 (6.4)	31.0 (18.5)	11.8 (7.8)	33.1 (19.4)	7.8 (5.9)	8.9 (5.7)

As in Task 1, the DIAMOND and SHORE-based approaches have better performances than the SHInterp method, indicating that DIAMOND and SHORE are more agnostic to scanner or protocol dependent noise/errors in the measurements. The LinearRISH approach still has the best performances in predicting the FA, MD, RISH and MD measures, though it has higher error in RTOP than SHInterp.

For CNN-based approaches, the simple SH-1Layer approach has better performances than SHResNetb1 to SHResNet-b4 and DIQT, implying that these complex networks need to be further trained to improve the perforamnces. Nonetheless, other deep-networks based methods, such as SHResNet-b0 and CMResNet-b, have better performances than SH-1Layer.

The global evaluation results for Task 2b are provided in Figs. S1 to S3 and Table S1 in the Supplementary Materials. Overall, similar results as in Task 2a were found for this task. The LinearRISH approach still has the best performances in most metrics but the RTOP measure. The DIAMOND-based methods and the SH-1Layer approach have similar performances. SHResNet-b0 and DIQT are two of the best CNN-based algorithms.

3.2.2. Regional evaluation

Table 5: Summary of the average percentage of APE values and two ROIs from the Desikan-Killiany atlas from FreeSurfer with the highest and lowest APE values for algorithms evaluated for Task 2a.

	APE (%)		ROIs with the highest APE	ROIs with the lowest APE
	GM	WM		
SHInterp	30.8	19.0	ctx-rh-postcentral, ctx-rh-superiorparietal	wm-rh-paracentral, wm-rh-parstriangularis
DIAMOND-a	22.8	15.3	ctx-rh-frontalpole, ctx-rh-superiorparietal	wm-lh-paracentral, wm-rh-paracentral
DIAMOND-b	22.8	15.2	ctx-rh-frontalpole, ctx-rh-superiorparietal	wm-lh-paracentral, wm-rh-paracentral
SHOREInterp	26.3	17.5	ctx-rh-superiorparietal, ctx-rh-postcentral	wm-rh-paracentral, wm-lh-paracentral
LinearRISH	12.7	8.6	ctx-rh-postcentral, ctx-rh-superiorparietal	wm-rh-paracentral, wm-lh-paracentral
SH-1Layer	22.9	13.6	ctx-lh-frontalpole, ctx-rh-inferiortemporal	wm-rh-paracentral, wm-lh-precentral
SHResNet-a	25.1	15.9	ctx-rh-superiorparietal, ctx-rh-postcentral	wm-rh-paracentral, wm-lh-paracentral
SHResNet-b0	18.2	13.0	ctx-lh-entorhinal, ctx-rh-parsorbitalis	wm-rh-paracentral, wm-rh-parsopercularis
SHResNet-b1	43.8	35.7	ctx-lh-inferiortemporal, ctx-rh-parsorbitalis	ctx-rh-caudalanteriorcing., ctx-rh-transversetemporal
SHResNet-b2	28.5	17.0	ctx-rh-postcentral, ctx-rh-transversetemporal	wm-rh-paracentral, wm-rh-insula
SHResNet-b3	32.2	20.0	ctx-rh-precentral, ctx-rh-postcentral	wm-rh-paracentral, wm-rh-rostralanteriorcing.
SHResNet-b4	23.1	14.6	ctx-rh-temporalpole, ctx-rh-unknown	wm-rh-paracentral, wm-rh-precentral
SphericalNet	24.9	15.8	ctx-lh-parsorbitalis, ctx-rh-superiorparietal	wm-rh-paracentral, wm-lh-paracentral
Tinypatch	24.2	20.1	wm-rh-entorhinal, wm-rh-entorhinal	wm-lh-transversetemporal, wm-lh-fusiform
DIQT	33.5	18.4	ctx-lh-parsorbitalis, ctx-rh-parsorbitalis	wm-rh-parsopercularis, wm-rh-supramarginal
CMResNet-b	19	12	ctx-rh-superiorparietal, ctx-rh-postcentral	wm-rh-paracentral, wm-lh-precentral

The spatial distribution of APE values has similar patterns as in Task 1. In particular, the frontal and the temporal lobes have relatively higher errors than other regions which may be caused by image distortions or head motions. A detailed illustration of the spatial patterns for the algorithms evaluated for Task 2a and Task 2b is provided in Figs. S4 and S5 in the Supplementary Materials. Table 5 shows the mean APE values in GM and WM and the two ROIs with the highest or lowest APE values for Task 2a. The corresponding results for Task 2b are shown in Table S2 in the Supplementary Materials.

The estimation errors have similar spatial patterns as in Task 1. In particular, the errors in gray matter are significantly higher than the errors in white matter. The cortical regions in the superiorparietal, the entorhinal, the temporalpole and the frontalpole have the highest APE values. On the other hand, white matter in paracentral areas consistently exhibits the lowest APE for most algorithms.

4. Discussion

In this work, we have evaluated the performance of 19 algorithms that have participated in an open competition on harmonization of multi-shell dMRI scans from different scanners and protocols. Next, we discuss several factors that may limit the results of this study and possible solutions to further improve the performance in future work.

4.1. Sample size of training data

The performance of harmonization algorithms may depend on the number of subjects used in training (Karayumak et al. 2019). Ideally, the number of subjects should be large enough so that

the training dataset contains sufficient information to learn the variability across datasets acquired using different scanners and/nor protocols. If the harmonization algorithm is based on deep networks, the number of training examples should be large enough to ensure the convergence of the training algorithms and to avoid overfitting. But, in practice, travelling subjects are expensive and logistically difficult, making them infeasible for multi-center studies. Thus, many studies only use one subject, or a phantom, to test the stability of imaging measures (Teipel et al. 2011). The benchmark database evaluated in this study is one of the largest open datasets with scans from traveling subjects using different scanners and protocols. However, the analysis results show that the performance of several algorithms with complex network structure is worse than a simple one-layer network, i.e. Sh-1Layer, indicating the training data is still insufficient for complex algorithms. Thus, more effective network structures and training algorithms need to be explored in future multi-site harmonization studies.

4.2. Inter-subject variability and adaptive prediction

In the evaluation results, the LinearRISH method has demonstrated better performance than other algorithms in most of the evaluation metrics, followed by the DIAMOND and SHORE-based approaches. Among other reasons, three possible factors that contribute to its superior performances are as follows: First, the simple linear-regression approach is able to avoid overfitting and provide robust data predictions. The voxel-wise linear models used in LinearRISH are able to reliably reduce space dependent variability in the datasets. Second, this approach adaptively selected different training subjects to predict each testing dataset based on the similarities in the corresponding Prisma-ST scans. Though it is expected that there exist subject-independent factors that are related to the cross-scanner and cross-protocol variability in the dataset, there may also exist other factors, such as head sizes and shapes, that lead to subject-specific variability across scans. The adaptive prediction approach used by LinearRISH was shown to be an effective solution to reduce subject-dependent variability in learning mappings between dMRI data from different scanners and protocols. However, the potential of adaptive prediction in clinical applications with pathological data need to be explored in future work. Third, LinearRISH is the only method that uses 6th order SH representations, while other methods use 4th order SH representation. The higher order SH representations may provide more detailed information on the data that is helpful to improve the performance of harmonization algorithms.

4.3. On evaluation metrics

This study used several metrics, including DTI, DKI, MAP-MRI and RISH features, to evaluate the performance of harmonization algorithms on a global and regional level. Note that the FA and MD measures were computed only using dMRI signals at the lower b-shell at b = 1200 s/mm^2, since at higher b-values higher-order cumulants become more prominent. Moreover, the RISH features were computed separately for the two b-shells at b = 1200, 3000 s/mm^2, respectively. For most algorithms, the prediction error of RISH features is higher for higher b-values. Moreover, the MK measure from DKI and the RTOP measure from MAP-MRI were computed by integrating dMRI signals at both b-shells. The two measures reflect different aspects of the signals. In particular, the MK measure was computed based on the cumulant expansion, which do not provide an accurate prediction of diffusion signals to high b-values. Moreover, the maximum b-value at b=3000 s/mm^2 might be beyond the radius of convergence for DKI, jeopardizing accuracy but potentially improving precision (Chuhutin et al. 2017). On the other hand, the RTOP measure was computed based on an analytical expression of the ensemble average propagator estimated using diffusion signals. Though other approaches were proposed to compute the RTOP measure, these methods all rely on continuous extrapolation of diffusion signals. Thus, the RTOP measure

is sensitive to the decay of diffusion signals and the estimation methods. Although at least two b-shells are needed to estimate the MK and RTOP for characterizing non-exponential signal decays, more b-values are expected to further improve the consistency of estimation results. In the evaluation results, the LinearRISH approach achieved the best performance in almost all metrics except RTOP which may be caused by errors in relative signal decays along the radial direction of b-values of the predicted signals. Caution should be placed in the results from the RISH (R0 and R2) metrics since these are based on the same underlying model as LinearRISH. However, the evaluation with different metrics should reduce potential bias in metrics whose underlying model is similar to the proposed algorithms. These diversified metrics could provide a guideline on the selection of harmonization algorithms corresponding to different metrics.

The ROI based analysis, e.g. Figure 7, shows that the GM has much higher APE values than WM. A possible reason is that GM has more significant heterogeneity due to different layers and cortical folds, which make it more difficult to harmonize the dMRI data. The findings in the regional analysis suggest that caution should be taken when interpreting results on these high-APE regions. Future work is needed to develop more reliable algorithms to harmonize these ROIs. Finally, we mention that a limitation of the analysis is that only the APE is used to compare different dMRI measures. The APE metric may not reflect the impact of the differences in dMRI data on statistical analysis in clinical studies. Further evaluation metrics will be needed in future studies to highlight the differences between dMRI data for clinical studies from different angles.

4.4. On signal representations and models

The harmonization algorithms used four types of approaches to represent or model diffusion signals including spherical harmonics (SH), Spherical Harmonics and a Radial Decomposition (SHARD), the DIAMOND model and the SHORE model. In particular, SH was the most commonly used approach that provides a complete basis for the representation of single-shell diffusion signals. Typically, fourth- or sixth-order SH representations were used to remove high-frequency noise. Moreover, the SH coefficients were usually separately estimated for different b-shells without modeling the radial signal decay. On the other hand, the novel SHARD representation and the representations based on the DIAMOND and SHORE models were computed using multi-shell diffusion signals. These approaches use either parsimonious/band-limited representations or biophysical models to remove noise in the signals. In the interpolation-based approaches, SH, DIAMOND and SHORE were all used to predict dMRI signals without removing cross-scanner and cross-protocol variability. It was interesting that all these approaches, especially the DIAMOND based methods, provided lower prediction error than the reference, indicating that these biophysical and statistical hybrid models accurately characterize the diffusion properties of the underlying tissues and reduce the variability across scanners, particularly variability caused by measurement noise.

5. Conclusion

In this study, we have presented a comprehensive evaluation of the performance of multi-shell harmonization algorithms for reducing cross-scanner and/or cross-protocol variability in dMRI data using a benchmark database. The results showed that some algorithms were able to significantly reduce the variability to a similar level as the scan-rescan variability between different scans of the same subjects using the same scanner and protocol. Moreover, the results from different types of algorithms indicate that inter-subject variability and measurement noise are important factors causing variability among dMRI scans. The performance of a large set of algorithms evaluated in this study could provide useful information to guide the selection

of harmonization algorithms in future multi-site studies with travelling subjects. The benchmark database used in this study can be obtained on the following webpage: https://www.cardiff.ac.uk/cardiff-university-brain-research-imaging-centre/research/projects/cross-scanner-and-cross-protocol-diffusion-MRI-data-harmonisation. Evaluation pipelines and the results are available at: https://projects.iq.harvard.edu/files/cdmri2018/files/mushac_evaluation.zip. The organizing team of MUSHAC can be contacted by email at cdmri18@cs.ucl.ac.uk.

Acknowledgements

CMWT is supported by a Rubicon grant (680-50-1527) from the Netherlands Organisation for Scientific Research (NWO) and Wellcome Trust grant (096646/Z/11/Z). This project has received funding under the European Union’s Horizon 2020 research and innovation programme under grant agreement No. 634541 and from Engineering and Physical Sciences Research Council (EPSRC EP/R006032/1, M020533/1), funding FG. DKJ were supported by MRC grant MR/K004360/1. Scan costs were supported by the National Centre for Mental Health (NCMH) with funds from Health and Care Support Wales and by the Wellcome Trust. JV is a Postdoctoral Fellow of the Research Foundation - Flanders (FWO; grant number 12S1615N). LN is supported in part by NIH grants R21MH115280, R21MH116352 and K01MH117346. SCK and YR are supported in part by NIH grant R01MH119222. AZ and JP have received funding from the European Union's Horizon 2020 research and innovation program under the Marie Sklodowska-Curie grant agreement No 765148. KS is supported in part by NIH grants R01EB017230, and T32EB001628. FS is supported in part by USC ADRC 5P50AG005142 and R01NS100973. EK acknowledges support from UK EPSRC EP/M020533/1, EP/N018702/1 and EU H2020 634541.

Supplementary material

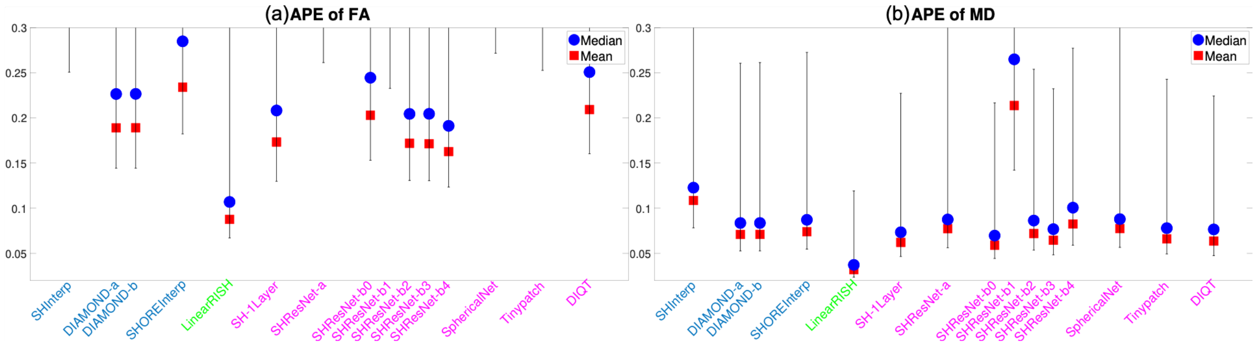

Figure S1: *Global APE values for FA and MD measures computed using diffusion signals at the b-shell with b = 1200 s/mm*^{2} *for algorithms evaluated for Task 2b.*

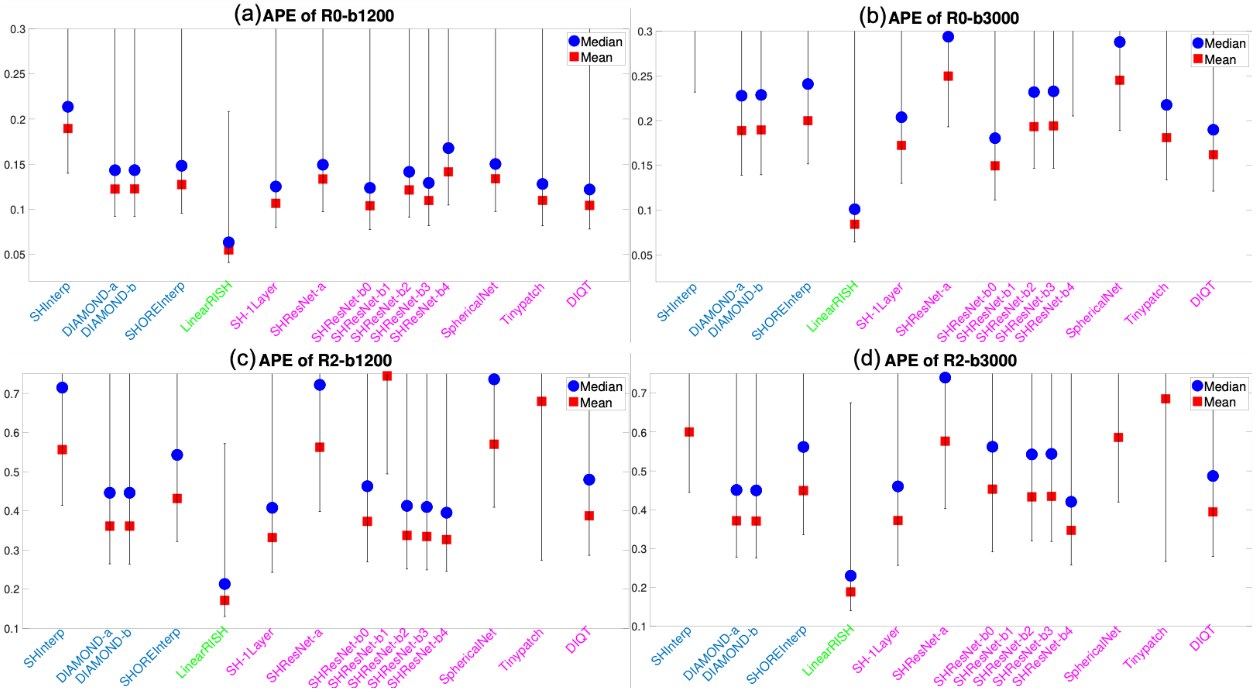

Figure S2: *Global APE values for the zeroth and second order RISH features, R0 and R2, of diffusion signals at two b-shells for the algorithms evaluated for Task 2b.*

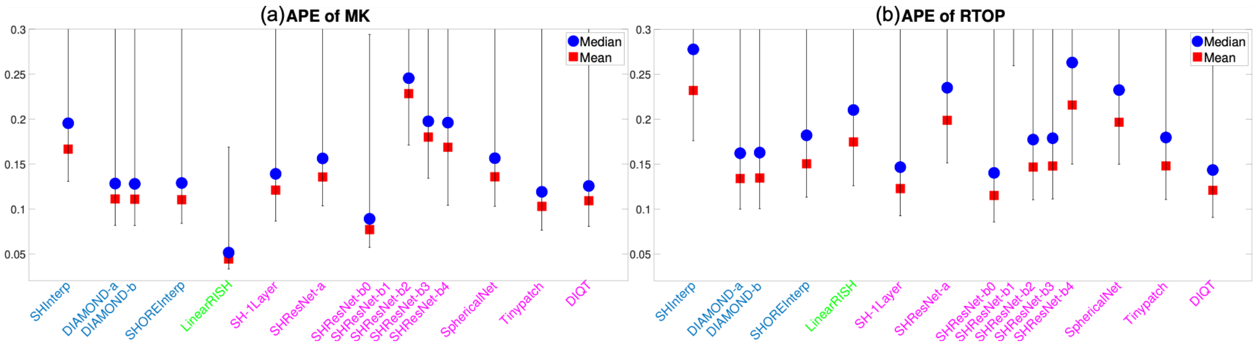

Figure S3: *Global APE values for the MK and RTOP measures computed using multi-shell diffusion signals for algorithms evaluated for Task 2b.*

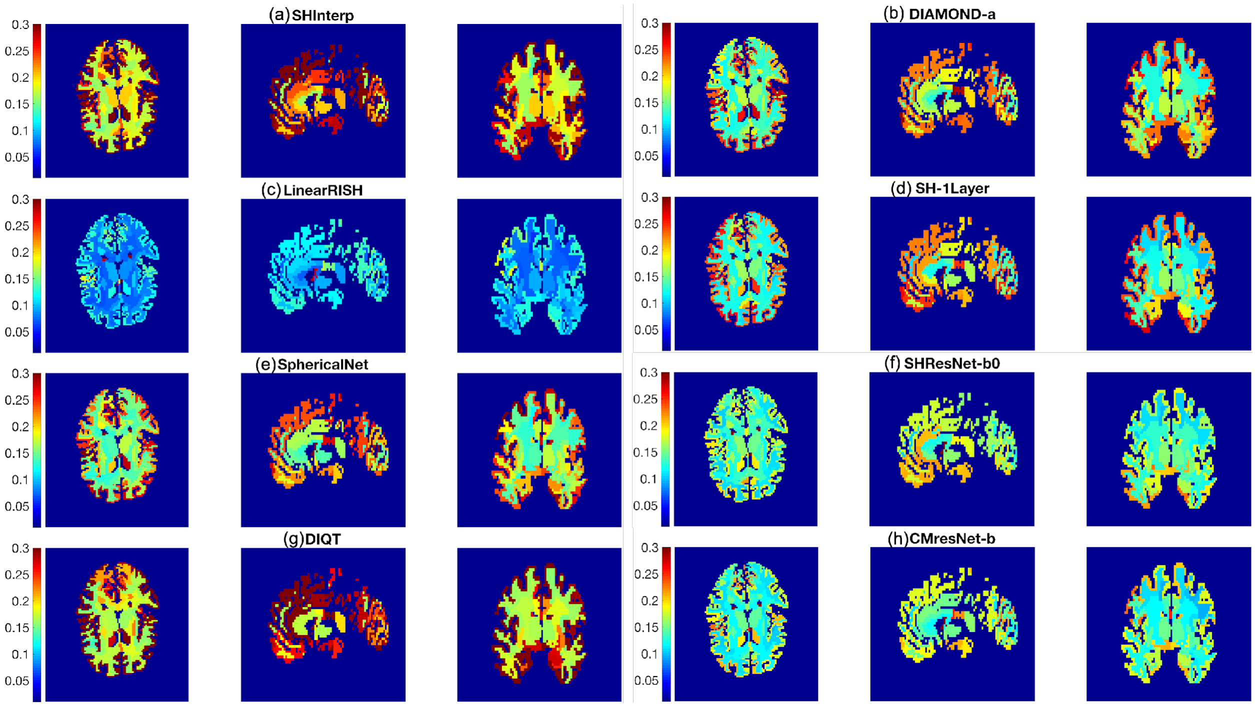

Figure S4: *Illustration of the average regional APE values in different brain regions defined by the Desikan-Killiany atlas from FreeSurfer for several representative algorithms evaluated for Task 2a.*

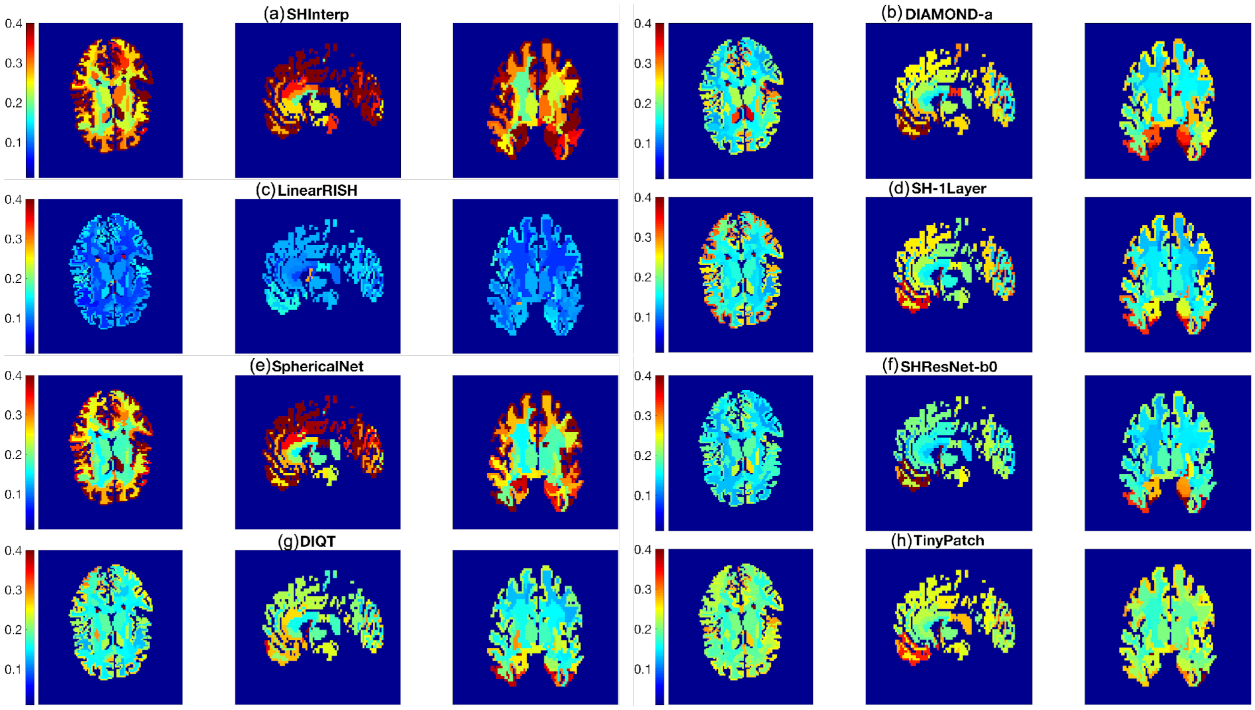

Figure S5: *Illustration of the average regional APE values of all measures in different brain regions defined by the Desikan-Killiany atlas from FreeSurfer for several representative algorithms evaluated for Task 2b.*

Table S1: *Summary of the percentage APE values, i.e. APEx100%, of reported measures in global evaluation for Task 2b.*

	APE (%): Mean (Standard deviation)							
	FA	MD	R0 (b=1200)	R2 (b=1200)	R0 (b=3000)	R2 (b=3000)	MK	RTOP
SHInterp	32.1 (20.6)	10.9 (7.8)	18.9 (13.9)	55.6 (29.7)	30.1 (20.2)	60.0 (33.3)	16.6 (11.8)	23.2 (15.1)
DIAMOND-a	18.7 (12.2)	6.8 (4.6)	11.8 (8.1)	35.7 (20.8)	20.7 (12.3)	37.1 (22.9)	11.6 (7.9)	14.6 (8.7)
DIAMOND-b	18.7 (12.3)	6.8 (4.6)	11.8 (8.1)	35.8 (20.8)	20.6 (12.3)	37.4 (23.2)	11.5 (7.9)	14.5 (8.7)
SHOREInterp	23.4 (15.1)	7.4 (4.9)	12.7 (8.8)	43.2 (24.4)	20.0 (12.8)	44.9 (25.8)	11.0 (7.7)	15.0 (9.5)
LinearRISH	8.8 (5.5)	3.2 (2.2)	5.5 (3.8)	17.1 (10.2)	8.4 (5.5)	18.9 (11.5)	4.4 (3.1)	17.5 (10.7)
SH-1Layer	17.3 (11.1)	6.2 (4.1)	10.7 (7.2)	33.2 (19.5)	17.2 (11.5)	37.2 (20.3)	12.1 (8.3)	12.3 (8.0)
SHResNet-a	33.9 (20.8)	7.7 (5.6)	13.3 (9.9)	56.3 (28.6)	25.0 (17.4)	57.6 (28.9)	13.6 (9.8)	19.9 (13.4)
SHResNet-b0	20.3 (13.0)	5.9 (4.0)	10.4 (6.8)	37.3 (21.2)	14.9 (9.4)	45.3 (22.8)	7.7 (5.4)	11.5 (7.0)
SHResNet-b1	31.9 (21.5)	21.4 (11.1)	47.4 (28.9)	74.5 (60.5)	49.2 (31.0)	167.6 (148.3	37.3 (27.0)	34.6 (22.6)
SHResNet-b2	17.2 (11.4)	7.2 (4.6)	12.1 (8.4)	33.7 (20.4)	19.3 (12.6)	43.3 (24.5)	22.8 (18.9)	14.7 (9.3)
SHResNet-b3	17.1 (11.3)	6.5 (4.2)	11.0 (7.3)	33.4 (20.2)	19.4 (12.6)	43.5 (24.4)	18.0 (14.1)	14.8 (9.3)
SHResNet-b4	16.3 (11.1)	8.3 (4.8)	14.1 (9.2)	32.7 (20.5)	30.7 (17.5)	34.7 (21.5)	16.9 (9.7)	21.6 (12.3)
SphericalNet	34.7 (21.3)	7.7 (5.6)	13.4 (9.9)	57.0 (28.9)	24.5 (17.1)	58.6 (29.4)	13.6 (9.7)	19.7 (13.3)
Tinypatch	45.5 (20.4)	6.6 (4.4)	11.0 (7.5)	68.0 (24.0)	18.1 (11.4)	68.5 (23.8)	10.3 (7.1)	14.8 (9.2)
DIQT	20.9 (13.8)	6.4 (4.0)	10.4 (7.1)	38.7 (22.6)	16.2 (11.0)	39.5 (22.3)	10.9 (7.7)	12.1 (8.0)

Table S2: *Summary of the average percentage APE values and two ROIs from the Desikan-Killiany atlas from FreeSurfer with the highest and lowest APE values for algorithms evaluated for Task 2b.*

	APE (%)		ROIs with the highest APE	ROIs with the lowest APE
	GM	WM		
SHInterp	43.9	30.7	ctx-rh-superiortemporal, ctx-lh-superiortemporal	wm-rh-paracentral, wm-lh-parstriangularis
DIAMOND-a	27.6	18.3	ctx-lh-entorhinal, ctx-rh-entorhinal	wm-rh-paracentral, wm-rh-parsopercularis
DIAMOND-b	27.6	18.2	ctx-lh-entorhinal, ctx-rh-entorhinal	wm-rh-paracentral, wm-rh-parsopercularis
SHOREInterp	30.0	20.8	ctx-lh-temporalpole, ctx-rh-medialorbitofrontal	wm-rh-paracentral, wm-rh-parstriangularis
LinearRISH	13.5	9.6	ctx-rh-postcental, ctx-rh-transversetemporal	wm-rh-paracentral, wm-rh-parstriangularis
SH-1Layer	27.1	16.7	ctx-rh-parsorbitalis, ctx-lh-entorhinal	wm-rh-paracentral, wm-lh-paracentral
SHResNet-a	39.3	25.5	ctx-lh-frontalpole, ctx-rh-superiorparietal	wm-rh-paracentral, wm-lh-insula
SHResNet-b0	22.8	16.4	ctx-lh-entorhinal, ctx-rh-medialorbitofrontal	wm-lh-inferiorparietal wm-lh-supramargina
SHResNet-b1	52.2	57.6	ctx-rh-middletemporal, ctx-rh-bankssts	ctx-lh-insula, wm-lh-entorhinal
SHResNet-b2	33.7	21.6	ctx-lh-temporalpole, ctx-lh-entorhinal	wm-rh-paracentral, wm-rh-parsopercularis
SHResNet-b3	32.4	20.2	ctx-lh-temporalpole, ctx-lh-medialorbitofrontal	wm-rh-paracentral, wm-rh-parstriangularis
SHResNet-b4	33.9	23.9	ctx-lh-frontalpole, ctx-rh-frontalpole	vm-lh-lateraloccipital, wm-rh-lateraloccipita
SphericalNet	38.7	25.1	ctx-lh-frontalpole, ctx-lh-superiorparietal	wm-rh-paracentral, wm-lh-insula
Tinypatch	25.9	20.6	ctx-lh-frontalpole, ctx-lh-inferiortemporal	wm-rh-transversetemporal, wm-lh-supramaginal
DIQT	25.9	16.6	ctx-lh-frontalpole, ctx-rh-inferiortemporal	wm-rh-paracentral, wm-lh-paracentral

Reference

Alexander, Daniel C., Darko Zikic, Aurobrata Ghosh, Ryutaro Tanno, Viktor Wottschel, Jiaying Zhang, Enrico Kaden, et al. 2017. “Image Quality Transfer and Applications in Diffusion MRI.” *NeuroImage* 152 (May): 283–98.

Andersson, Jesper L. R., Stefan Skare, and John Ashburner. 2003. “How to Correct Susceptibility Distortions in Spin-Echo Echo-Planar Images: Application to Diffusion Tensor Imaging.” *NeuroImage* 20 (2): 870–88.

Andersson, Jesper L. R., and Stamatios N. Sotiropoulos. 2016. “An Integrated Approach to Correction for off-Resonance Effects and Subject Movement in Diffusion MR Imaging.” *NeuroImage* 125 (January): 1063–78.

Asman, A. J., and B. A. Landman. 2014. “Hierarchical Performance Estimation in the Statistical Label Fusion Framework.” Medical Image Analysis 18(7): 1070-1081.

Avants, Brian B., Nicholas J. Tustison, Gang Song, Philip A. Cook, Arno Klein, and James C. Gee. 2011. “A Reproducible Evaluation of ANTs Similarity Metric Performance in Brain Image Registration.” *NeuroImage* 54 (3): 2033–44.

Bammer, R., M. Markl, A. Barnett, B. Acar, M. T. Alley, N. J. Pelc, G. H. Glover, and M. E. Moseley. 2003. “Analysis and Generalized Correction of the Effect of Spatial Gradient Field Distortions in Diffusion-Weighted Imaging.” *Magnetic Resonance in Medicine*. https://doi.org/10.1002/mrm.10545.

Blumberg, S. B., M. Palombo, C. S. Khoo, C. M. W. Tax, R. Tanno, and D. C. Alexander. 2019. “Multi-Stage Prediction Networks for Data Harmonisation.” In *Medical Image Computing and Computer-Assisted Intervention – MICCAI 2019*, 411-419.

Blumberg, Stefano B., Ryutaro Tanno, Iasonas Kokkinos, and Daniel C. Alexander. 2018. “Deeper Image Quality Transfer: Training Low-Memory Neural Networks for 3D Images.” In *Medical Image Computing and Computer Assisted Intervention – MICCAI 2018*, 118–25. Springer International Publishing.

Boekel, W., B. U. Forstmann, and M. C. Keuken. 2017. “A Test-Retest Reliability Analysis of Diffusion Measures of White Matter Tracts Relevant for Cognitive Control.” *Psychophysiology*. https://doi.org/10.1111/psyp.12769.

Breiman, L. 2001. “Random forests.” Machine Learning 45(1): 5-32.

Cardoso, M. Jorge, Marc Modat, Robin Wolz, Andrew Melbourne, David Cash, Daniel Rueckert, and Sebastien Ourselin. 2015. “Geodesic Information Flows: Spatially-Variant Graphs and Their Application to Segmentation and Fusion.” *IEEE Transactions on Medical Imaging* 34 (9): 1976–88.

Cetin Karayumak, Suheyla, Sylvain Bouix, Lipeng Ning, Anthony James, Tim Crow, Martha Shenton, Marek Kubicki, and Yogesh Rathi. 2019. “Retrospective Harmonization of Multi-Site Diffusion MRI Data Acquired with Different Acquisition Parameters.” *NeuroImage* 184 (January): 180–200.

Cheng, Jian, Tianzi Jiang, and Rachid Deriche. 2011. “Theoretical Analysis and Practical Insights on Eap Estimation via a Unified Hardi Framework.” In *MICCAI Workshop on Computational Diffusion MRI (CDMRI)*. https://hal.inria.fr/inria-00615430/.

Christiaens, Daan, Lucilio Cordero-Grande, Jana Hutter, Anthony N. Price, Maria Deprez, Joseph V. Hajnal, and J-Donald Tournier. 2018. “Learning Compact Q-Space Representations for Multi-Shell Diffusion-Weighted MRI.” *IEEE Transactions on Medical Imaging*, October. https://doi.org/10.1109/TMI.2018.2873736.

Duan, Fei, Tengda Zhao, Yong He, and Ni Shu. 2015. “Test-Retest Reliability of Diffusion

Measures in Cerebral White Matter: A Multiband Diffusion MRI Study." *Journal of Magnetic Resonance Imaging*. https://doi.org/10.1002/jmri.24859.
Fischl, Bruce. 2012. "FreeSurfer." *NeuroImage 62(2): 774-781*.
Fortin, Jean-Philippe, Drew Parker, Birkan Tunç, Takanori Watanabe, Mark A. Elliott, Kosha Ruparel, David R. Roalf, et al. 2017. "Harmonization of Multi-Site Diffusion Tensor Imaging Data." *NeuroImage* 161 (November): 149–70.
Garyfallidis, Eleftherios, Matthew Brett, Bagrat Amirbekian, Ariel Rokem, Stefan van der Walt, Maxime Descoteaux, Ian Nimmo-Smith, and Dipy Contributors. 2014. "Dipy, a Library for the Analysis of Diffusion MRI Data." *Frontiers in Neuroinformatics* 8 (February): 8.
Glasser, Matthew F., Stamatios N. Sotiropoulos, J. Anthony Wilson, Timothy S. Coalson, Bruce Fischl, Jesper L. Andersson, Junqian Xu, et al. 2013. "The Minimal Preprocessing Pipelines for the Human Connectome Project." *NeuroImage* 80 (October): 105–24.
Grech-Sollars, Matthew, Patrick W. Hales, Keiko Miyazaki, Felix Raschke, Daniel Rodriguez, Martin Wilson, Simrandip K. Gill, et al. 2015. "Multi-Centre Reproducibility of Diffusion MRI Parameters for Clinical Sequences in the Brain." *NMR in Biomedicine* 28 (4): 468–85.
He, Kaiming, Xiangyu Zhang, Shaoqing Ren, and Jian Sun. 2016. "Deep Residual Learning for Image Recognition." In *Proceedings of the IEEE Conference on Computer Vision and Pattern Recognition*, 770–78.
Hu, Jie, Li Shen, Samuel Albanie, Gang Sun, and Enhua Wu. 2017. "Squeeze-and-Excitation Networks." *arXiv [cs.CV]*. arXiv. http://arxiv.org/abs/1709.01507.
Huo, Yuankai, Zhoubing Xu, Katherine Aboud, Prasanna Parvathaneni, Shunxing Bao, Camilo Bermudez, Susan M. Resnick, Laurie E. Cutting, and Bennett A. Landman. 2018. "Spatially Localized Atlas Network Tiles Enables 3D Whole Brain Segmentation from Limited Data." In *Medical Image Computing and Computer Assisted Intervention – MICCAI 2018*, 698–705. Springer International Publishing.
Jenkinson, Mark, Christian F. Beckmann, Timothy E. J. Behrens, Mark W. Woolrich, and Stephen M. Smith. 2012. "FSL." *NeuroImage*. https://doi.org/10.1016/j.neuroimage.2011.09.015.
Jenkinson, Mark, Mickael Pechaud, Stephen Smith, and Others. 2005. "BET2: MR-Based Estimation of Brain, Skull and Scalp Surfaces." In *Eleventh Annual Meeting of the Organization for Human Brain Mapping*, 17:167. Toronto.
Jenkinson, M. and S.M Smith. 2001. "A global optimisation method for robust affine registration of brani images." *Medical Image Analysis 5(2)*: 143-156.
Jensen, Jens H., Joseph A. Helpern, Anita Ramani, Hanzhang Lu, and Kyle Kaczynski. 2005. "Diffusional Kurtosis Imaging: The Quantification of Non-Gaussian Water Diffusion by Means of Magnetic Resonance Imaging." *Magnetic Resonance in Medicine: Official Journal of the Society of Magnetic Resonance in Medicine / Society of Magnetic Resonance in Medicine* 53 (6): 1432–40.
Jones, D.K., D.C. Alexander, R. Bowtell, M. Cercignani, F. Dell'Acqua, D.J. McHugh, K.L. Miller, M. Palombo, G.J.M Parker, U.S. Rudrapatna, C.M.W. Tax. 2018. "Microstructural imaging of the human brain with a 'super-scanner': 10 key advantages of ultra-strong gradients for diffusion MRI." *NeuroImage, 182:8-38.*
Kellner, Elias, Bibek Dhital, Valerij G. Kiselev, and Marco Reisert. 2016. "Gibbs-Ringing Artifact Removal Based on Local Subvoxel-Shifts." *Magnetic Resonance in Medicine 76(5): 1574-1581*.
Klein, A, Andersson J, Ardekani BA, Ashburner J, Avants B, Chiang MC, Christensen GE, Collins DL, Gee J, Hellier P, Song JH, Jenkinson M, Lepage C, Rueckert D, Thompson P, Vercauteren T, Woods RP, Mann JJ, Parsey RV. 2009. "Evaluation of 14 nonlinear deformation algorithms applied to human brain MRI registration." NeuroImage 46(3): 786-802.
Koppers, Simon, Luke Bloy, Jeffrey I. Berman, Chantal M. W. Tax, J. Christopher Edgar, and

Dorit Merhof. 2018. “Spherical Harmonic Residual Network for Diffusion Signal Harmonization.” *arXiv [cs.CV]*. arXiv. http://arxiv.org/abs/1808.01595.
Koppers, Simon, and Dorit Merhof. 2018. “DELIMIT PyTorch - An Extension for Deep Learning in Diffusion Imaging.” *arXiv [cs.LG]*. arXiv. http://arxiv.org/abs/1808.01517.
Kubicki, Marek, Robert McCarley, Carl-Fredrik Westin, Hae-Jeong Park, Stephan Maier, Ron Kikinis, Ferenc A. Jolesz, and Martha E. Shenton. 2007. “A Review of Diffusion Tensor Imaging Studies in Schizophrenia.” *Journal of Psychiatric Research* 41 (1-2): 15–30.
Landman, Bennett A., Alan J. Huang, Aliya Gifford, Deepti S. Vikram, Issel Anne L. Lim, Jonathan A. D. Farrell, John A. Bogovic, et al. 2011. “Multi-Parametric Neuroimaging Reproducibility: A 3-T Resource Study.” *NeuroImage* 54 (4): 2854–66.
Liu, Ming-Yu, Thomas Breuel, and Jan Kautz. 2017. “Unsupervised Image-to-Image Translation Networks.” *arXiv [cs.CV]*. arXiv. http://arxiv.org/abs/1703.00848.
Meijering, Erik H. W., Wiro J. Niessen, Josien P. W. Pluim, and Max A. Viergever. 1999. “Quantitative Comparison of Sinc-Approximating Kernels for Medical Image Interpolation.” In *Medical Image Computing and Computer-Assisted Intervention – MICCAI’99*, 210–17. Springer Berlin Heidelberg.
Merlet, Sylvain L., and Rachid Deriche. 2013. “Continuous Diffusion Signal, EAP and ODF Estimation via Compressive Sensing in Diffusion MRI.” *Medical Image Analysis* 17 (5): 556–72.
Mirzaalian, H., L. Ning, P. Savadjiev, O. Pasternak, S. Bouix, O. Michailovich, S. Karmacharya, G. Grant, C.E. Marx, R.A. Morey, L.A. Flashman, M.S. George, T.W. McAllister, N. Andaluz, L. Shutter, R. Coimbra, R.D. Zafonte, M.J. Coleman, M. Kubicki, C.-F. Westin, M.B. Stein, M.E. Shenton, Y. Rathi, 2018. “Multi-Site Harmonization of Diffusion MRI Data in a Registration Framework.” *Brain Imaging and Behavior* 12 (1): 284–95.
Mirzaalian, H., L. Ning, P. Savadjiev, O. Pasternak, S. Bouix, O. Michailovich, G. Grant, C.E. Marx, R.A. Morey, L.A. Flashman, M.S. George, T.W. McAllister, N. Andaluz, L. Shutter, R. Coimbra, R.D. Zafonte, M.J. Coleman, M. Kubicki, C.-F. Westin, M.B. Stein, M.E. Shenton, Y. Rathi, 2016. “Inter-Site and Inter-Scanner Diffusion MRI Data Harmonization.” *NeuroImage* 135 (July): 311–23.
Moyer, D., G. Ver Steeg, C.M.W. Tax, P.M. Thompson, 2020. “Scanner invariant representations for diffusion MRI harmonization.” Magnetic Resonance in Medicine.
Mueller, Susanne G., Michael W. Weiner, Leon J. Thal, Ronald C. Petersen, Clifford R. Jack, William Jagust, John Q. Trojanowski, Arthur W. Toga, and Laurel Beckett. 2005. “Ways toward an Early Diagnosis in Alzheimer’s Disease: The Alzheimer’s Disease Neuroimaging Initiative (ADNI).” *Alzheimer’s & Dementia*. https://doi.org/10.1016/j.jalz.2005.06.003.
Nath, W., I. Lyu, K.G. Schilling, P. Parvathaneni, C. B. Hansen, Y. Tang, Y. Huo, V. A. Janve, Yurui Gao, I. Stepniewska, A. W. Anderson, B. A. Landman. 2019. “Enabling Multi-shell b-Value Generalizability of Data-Driven Diffusion Models with Deep SHORE.” in *Medical Image Computing and Computer-Assisted Intervention – MICCAI’19*.
Ning, Lipeng, Elisenda Bonet-Carne, Francesco Grussu, Farshid Sepehrband, Enrico Kaden, Jelle Veraart, Stefano B. Blumberg, et al. 2019. “Muti-Shell Diffusion MRI Harmonization and Enhancement Challenge (MUSHAC): Progress and Results.” In *Computational Diffusion MRI*, 217–24. Springer International Publishing.
Ning, Lipeng, Carl-Fredrik Westin, and Yogesh Rathi. 2015. “Estimating Diffusion Propagator and Its Moments Using Directional Radial Basis Functions.” *IEEE Transactions on Medical Imaging* 34 (10): 2058–78.
Özarslan, Evren, Cheng Guan Koay, Timothy M. Shepherd, Michal E. Komlosh, M. Okan İrfanoğlu, Carlo Pierpaoli, and Peter J. Basser. 2013. “Mean Apparent Propagator (MAP) MRI: A Novel Diffusion Imaging Method for Mapping Tissue Microstructure.” *NeuroImage* 78 (September): 16–32.
Pasternak, Ofer, Sinead Kelly, Valerie J. Sydnor, and Martha E. Shenton. 2018. “Advances in

Microstructural Diffusion Neuroimaging for Psychiatric Disorders.” *NeuroImage* 182 (November): 259–82.
Pedregosa, F., G. Varoquaux, A. Gramfort, V. Michel, B. Thirion, O. Grisel, M. Blondel, P. Prettenhofer, R. Weiss, V. Dubourg, J. Vanderplas, A. Passos, D. Cournapeau, M. Brucher, M. Perrot, E. Duchesnay. 2011. “Scikit-learn: Machine Learning in Python.” Journal of Machine Learning Research 12: 2825-2803.
Pohl, Kilian M., Edith V. Sullivan, Torsten Rohlfing, Weiwei Chu, Dongjin Kwon, B. Nolan Nichols, Yong Zhang, et al. 2016. “Harmonizing DTI Measurements across Scanners to Examine the Development of White Matter Microstructure in 803 Adolescents of the NCANDA Study.” *NeuroImage* 130 (April): 194–213.
Raffelt, David, J-Donald Tournier, Jurgen Fripp, Stuart Crozier, Alan Connelly, and Olivier Salvado. 2011. “Symmetric Diffeomorphic Registration of Fibre Orientation Distributions.” *NeuroImage* 56 (3): 1171–80.
Rudrapatna, S. U., G. D. Parker, J. Roberts, and D. K. Jones. 2018. “Can We Correct for Interactions between Subject Motion and Gradient-Nonlinearity in Diffusion MRI.” *Proceedings of the International Society for Magnetic Resonance in Medicine ... Scientific Meeting and Exhibition. International Society for Magnetic Resonance in Medicine. Scientific Meeting and Exhibition*.
Setsompop, K., R. Kimmlingen, E. Eberlein, T. Witzel, J. Cohen-Adad, J.A. McNab, B. Keil, M.D. Tisdall, P. Hoecht, P. Dietz, S.F. Cauley, V. Tountcheva, V. Matschl, V.H. Lenz, K. Heberlein, A. Potthast, H. Thein, J. Van Horn, A. Toga, F. Schmitt, D. Lehne, B.R. Rosen, V. Wedeen, L.L. Wald. 2013. “Pushing the limits of in vivo diffusion MRI for the Human Connectome Project.” NeuroImage. 80:220-233.
Scherrer, Benoit, Armin Schwartzman, Maxime Taquet, Mustafa Sahin, Sanjay P. Prabhu, and Simon K. Warfield. 2016. “Characterizing Brain Tissue by Assessment of the Distribution of Anisotropic Microstructural Environments in Diffusion-Compartment Imaging (DIAMOND).” *Magnetic Resonance in Medicine*. https://doi.org/10.1002/mrm.26372.
Scherrer, Benoit, Maxime Taquet, Armin Schwartzman, Etienne St-Onge, Gaetan Rensonnet, Sanjay P. Prabhu, and Simon K. Warfield. 2017. “Decoupling Axial and Radial Tissue Heterogeneity in Diffusion Compartment Imaging.” In *Information Processing in Medical Imaging*, 440–52. Springer International Publishing.
Shahim, Pashtun, Laurena Holleran, Joong H. Kim, and David L. Brody. 2017. “Test-Retest Reliability of High Spatial Resolution Diffusion Tensor and Diffusion Kurtosis Imaging.” *Scientific Reports* 7 (1): 11141.
Sotiropoulos, Stamatios N., Saad Jbabdi, Junqian Xu, Jesper L. Andersson, Steen Moeller, Edward J. Auerbach, Matthew F. Glasser, et al. 2013. “Advances in Diffusion MRI Acquisition and Processing in the Human Connectome Project.” *NeuroImage* 80 (October): 125–43.
Tax, Chantal MW, Francesco Grussu, Enrico Kaden, Lipeng Ning, Umesh Rudrapatna, C. John Evans, Samuel St-Jean, et al. 2019. “Cross-Scanner and Cross-Protocol Diffusion MRI Data Harmonisation: A Benchmark Database and Evaluation of Algorithms.” *NeuroImage* 195 (July): 285–99.
Teipel, Stefan J., Sigrid Reuter, Bram Stieltjes, Julio Acosta-Cabronero, Ulrike Ernemann, Andreas Fellgiebel, Massimo Filippi, Giovanni Frisoni, Frank Hentschel, Frank Jessen, Stefan Klöppel, Thomas Meindl, Petra J W Pouwels, Karl-Heinz Hauenstein, Harald Hampel. 2011. “Multicenter stability of diffusion tensor imaging measures: A European clinical and physical phantom study.” Psychiatry Research: Neuroimaging 194(30): 363-371.
Tournier, J-Donald, Fernando Calamante, David G. Gadian, and Alan Connelly. 2004. “Direct Estimation of the Fiber Orientation Density Function from Diffusion-Weighted MRI Data Using Spherical Deconvolution.” *NeuroImage* 23 (3): 1176–85.

Tustison, Nicholas J., Brian B. Avants, Philip A. Cook, Yuanjie Zheng, Alexander Egan, Paul A. Yushkevich, and James C. Gee. 2010. “N4ITK: Improved N3 Bias Correction.” *IEEE Transactions on Medical Imaging* 29 (6): 1310–20.

Veenith, Tonny V., Eleanor Carter, Julia Grossac, Virginia F. J. Newcombe, Joanne G. Outtrim, Victoria Lupson, Guy B. Williams, David K. Menon, and Jonathan P. Coles. 2013. “Inter Subject Variability and Reproducibility of Diffusion Tensor Imaging within and between Different Imaging Sessions.” *PloS One* 8 (6): e65941.

Veraart, Jelle, Els Fieremans, and Dmitry S. Novikov. 2016. “Diffusion MRI Noise Mapping Using Random Matrix Theory.” *Magnetic Resonance in Medicine: Official Journal of the Society of Magnetic Resonance in Medicine / Society of Magnetic Resonance in Medicine* 76 (5): 1582–93.

Vollmar, Christian, Jonathan O’Muircheartaigh, Gareth J. Barker, Mark R. Symms, Pamela Thompson, Veena Kumari, John S. Duncan, Mark P. Richardson, and Matthias J. Koepp. 2010. “Identical, but Not the Same: Intra-Site and Inter-Site Reproducibility of Fractional Anisotropy Measures on Two 3.0T Scanners.” *NeuroImage* 51 (4): 1384–94.

Zhang, Y., M. Brady, and S. Smith. 2001. “Segmentation of Brain MR Images through a Hidden Markov Random Field Model and the Expectation-Maximization Algorithm.” *IEEE Transactions on Medical Imaging* 20 (1): 45–57.

Zhou, Tinghui, Philipp Krähenbühl, Mathieu Aubry, Qixing Huang, and Alexei A. Efros. 2016. “Learning Dense Correspondence via 3D-Guided Cycle Consistency.” *arXiv [cs.CV]*. arXiv. http://arxiv.org/abs/1604.05383.

Zhu, Tong, Rui Hu, Xing Qiu, Michael Taylor, Yuen Tso, Constantin Yiannoutsos, Bradford Navia, et al. 2011. “Quantification of Accuracy and Precision of Multi-Center DTI Measurements: A Diffusion Phantom and Human Brain Study.” *NeuroImage* 56 (3): 1398–1411.
